# Melatonin enhances radiofrequency-induced NK antitumor immunity, causing cancer metabolism reprogramming and inhibition of multiple pulmonary tumor development

**DOI:** 10.1038/s41392-021-00745-7

**Published:** 2021-09-01

**Authors:** Ming Li, Bingjie Hao, Menghuan Zhang, Russel J. Reiter, Shumeng Lin, Tiansheng Zheng, Xiangyun Chen, Yanbei Ren, Liduo Yue, Baigenzhin Abay, Guojie Chen, Xiao Xu, Yufeng Shi, Lihong Fan

**Affiliations:** 1 Department of Respiratory Medicine, Shanghai Tenth People’s Hospital, Tongji University School of Medicine, Shanghai, China; 2 Institute of Energy Metabolism and Health, Shanghai Tenth People’s Hospital, Tongji University School of Medicine, Shanghai, China; 3 Tongji University Cancer Center, Shanghai Tenth People’s Hospital of Tongji University, School of Medicine, Tongji University, Shanghai, China; 4 Clinical Center For Brain And Spinal Cord Research, Tongji University, Shanghai, China; 5 Department of Cell Systems and Anatomy, University of Texas Health San Antonio, San Antonio, Texas USA; 6 National Scientific Medical Research Center, Astana, Kazakhstan; 7 State Key Laboratory of Molecular Biology, Shanghai Institute of Biochemistry and Cell Biology, Center for Excellence in Molecular Cell Science, Chinese Academy of Sciences, Shanghai, China

**Keywords:** Lung cancer, Lung cancer

## Abstract

Surgery is the common treatment for early lung cancer with multiple pulmonary nodules, but it is often accompanied by the problem of significant malignancy of other nodules in non-therapeutic areas. In this study, we found that a combined treatment of local radiofrequency ablation (RFA) and melatonin (MLT) greatly improved clinical outcomes for early lung cancer patients with multiple pulmonary nodules by minimizing lung function injury and reducing the probability of malignant transformation or enlargement of nodules in non-ablated areas. Mechanically, as demonstrated in an associated mouse lung tumor model, RFA not only effectively remove treated tumors but also stimulate antitumor immunity, which could inhibit tumor growth in non-ablated areas. MLT enhanced RFA-stimulated NK activity and exerted synergistic antitumor effects with RFA. Transcriptomics and proteomics analyses of residual tumor tissues revealed enhanced oxidative phosphorylation and reduced acidification as well as hypoxia in the tumor microenvironment, which suggests reprogrammed tumor metabolism after combined treatment with RFA and MLT. Analysis of residual tumor further revealed the depressed activity of MAPK, NF-kappa B, Wnt, and Hedgehog pathways and upregulated P53 pathway in tumors, which was in line with the inhibited tumor growth. Combined RFA and MLT treatment also reversed the Warburg effect and decreased tumor malignancy. These findings thus demonstrated that combined treatment of RFA and MLT effectively inhibited the malignancy of non-ablated nodules and provided an innovative non-invasive strategy for treating early lung tumors with multiple pulmonary nodules. Trial registration: www.chictr.org.cn, identifier ChiCTR2100042695, http://www.chictr.org.cn/showproj.aspx?proj=120931.

## Introduction

Lung cancer is the leading cause of cancer-related deaths worldwide; timely diagnosis and improved treatments are keys to improve the survival rate for this disease.^1,2^ With recent rapid advances in medical technology including computerized tomography (CT) screening, detection of early lung cancers has been greatly improved. Patients with early lung cancer often were associated with pulmonary ground-glass nodules (GGNs).^3–5^ For early lung cancer with multiple GGNs, the current standard protocol is recommended as following: if the main nodule diameter is ≥8 mm, it is suggested to remove the main lesion and follow-up for the other lesions. If the main nodule diameter is <8 mm, patients were taken for follow-up.^6^

A common challenge for patients with multiple GGNs is the development or malignant transformation of remaining nodules after surgery. In Asia, it was reported that about 21% of patients would experience enlarged GGNs or malignant transformation 2 years after their initial GGN surgery. It was quite often that, due to reduced lung function and other complications, patients with initial surgery were not compatible with an additional one.^7,8^ Therefore, a new innovative strategy for treating multiple GGNs is urgently needed.

Radiofrequency ablation (RFA) alone is recommended by the National Comprehensive Cancer Network guidelines for the treatment of early lung cancer; this treatment effectively removes the local tumor tissue with limited lung damages.^9^ Although RFA could induce antitumor immunity, it is still accompanied by high recurrence rates in the treatment of early lung cancer with multiple GGNs.^10^ Recently, combinations of local treatments with systemic therapy, such as targeted therapy, chemotherapy, and immunotherapy, have been suggested for reducing lung cancer recurrence and malignancy.^11–13^ However, these treatments have significant side effects or need proper diagnosis, which makes systemic therapies unsuitable for treating early lung cancer with GGNs. It is thus essential to devise a more effective and innovative treatment for early lung cancer patients with GGNs.

Melatonin (MLT) is an endogenously produced molecule that influences circadian rhythms and the cellular redox status.^14–16^ It is an important immunomodulatory molecule and exhibits an inhibitory effect on growth of certain cancers.^17–19^ Recent studies by Reiter et al. have proposed that MLT’s efficacy in inhibiting several cancer types was related to its ability to reverse Warburg metabolism in tumors.^20^

In this study, we provided data from patients and mouse lung tumor model, which presented that combined treatment of RFA and MLT not only profoundly destroyed lung tumor tissue in the ablation area but also inhibited the growth of lung cancer in non-ablated areas with limited lung function injury. Detailed analysis revealed that RFA efficiently eliminated local tumors and at the same time initiated antitumor immunity of natural killer (NK) cells. MLT further promoted RFA-induced NK antitumor immunity, causing cancer metabolism reprogram, and exerted synergistic inhibitory activity with RFA on lung tumor growth. Thus, these findings informed that the combined treatment of RFA and MLT strongly inhibited the growth of lung cancer in non-ablated areas and provided an innovative, efficient, and non-invasive local and systemic treatment for clinical care of early lung cancer patients with multiple GGNs.

## Results

### Combined treatment with RFA and MLT improves clinical outcome of early lung cancer patients with GGNs comparing to standard care (surgery)

From Jan 2018 to Jun 2018, a total of 42 early lung cancer patients with multiple GGNs who met the inclusion criteria were recruited into the study to evaluate the combined treatment with RFA and MLT. In this study, 21 patients received combined treatment with RFA and MLT (RFA + MLT group), while 21 patients were given standard care (Control group). As summarized in Table 1, the age, gender, pathology, and disease conditions for patients in the RFA + MLT and Control groups were carefully selected and then grouped. Thirteen women (61.90%) in the RFA + MLT group with the average age of 58.48 years (38–82 years) and 13 women (61.90%) in the Control group with average age of 58.47 years (36–78 years) were included. Cigarette smoking is an established risk factor for lung cancer, we thus considered this when grouping patients, 3 cases (14.29%) had comparable smoking history in the RFA + MLT and Control groups, respectively. Besides, the mean diameter of GGNs in patients of the RFA + MLT group was 0.88 cm (±0.22), and this for patients in the surgery group (Control) was 0.89 cm (±0.17) based on CT scan diagnosis. With regard to pulmonary nodule morphology, there were 13 cases of pure ground-glass opacities (pGGO) and 8 cases of mixed ground-glass opacities (mGGO) for the RFA + MLT group, and there were 15 cases in pGGO and 6 cases in mGGO for the Control group. In terms of pathology, such as atypical adenomatous hyperplasia (AAH) and adenocarcinoma in situ (AIS), 10 cases AAH and 11 cases AIS were recruited in both the RFA + MLT and Control groups. Of the 42 patients with multiple GGNs recruited into this study, 21 patients in the RFA + MLT group took MLT orally for 1 year.

**Table 1:** Baseline characteristics of the patient population

Characteristics	RFA+MLT (*n* = 21)	Surgery (*n* = 21)	*P* value
Age (years)			
Mean (SD)	58.48 (10.96)	58.47 (11.36)	0.9977
Range (years)	38–82	36–78	—
Gender (*n*, %)			1
Men	8 (38.10)	8 (38.10)	
Women	13 (61.90)	13 (61.90)	
Smoking (*n*, %)	3 (14.29)	3 (14.29)	1
Position (*n*, %)			
R.U.L.	9 (42.86)	7 (33.33)	0.2389
R.M.L.	1 (4.76)	1 (4.76)	
R.L.L.	3 (14.29)	5 (23.81)	
L.U.L.	6 (28.57)	2 (9.52)	
L.L.L.	2 (9.52)	6 (28.57)	
Dominant tumor size (cm)			
Mean	0.88 (0.22)	0.89 (0.17)	0.9028
Range (cm)	0.7–1.3	0.7–1.2	
Dominant tumor feature (*n*, %)			0.5178
pGGO	13 (61.90)	15 (71.43)	
mGGO	8 (38.10)	6 (28.57)	
Spicule sign (*n*, %)			0.6825
With	3 (14.29)	4 (19.05)	
None	18 (85.71)	17 (80.95)	
Pathology (*n*, %)			1
AAH	10 (47.62)	10 (47.62)	
AIS	11 (52.38)	11 (52.38)	
Lung function			
FEV_1_ (L)	2.61 (0.85)	2.39 (0.72)	0.3709
FEV_1_ measured/anticipated %	96.26 (16.36)	88.14 (7.26)	0.0441
VCMAX (L)	3.16 (0.90)	2.83 (0.61)	0.1719
VCMAX measured/anticipated %	93.95 (12.31)	88.20 (8.58)	0.0867

*SD* standard deviation, *pGGO* pure ground-glass opacities, *mGGO* mixed ground-glass opacities, *AAH* atypical adenomatous hyperplasia, *AIS* adenocariconoma in situ, *FEV*_*1*_ forced expiratory volume in 1 s, *VCMAX* maximal vital capacity

**Fig. 1:**
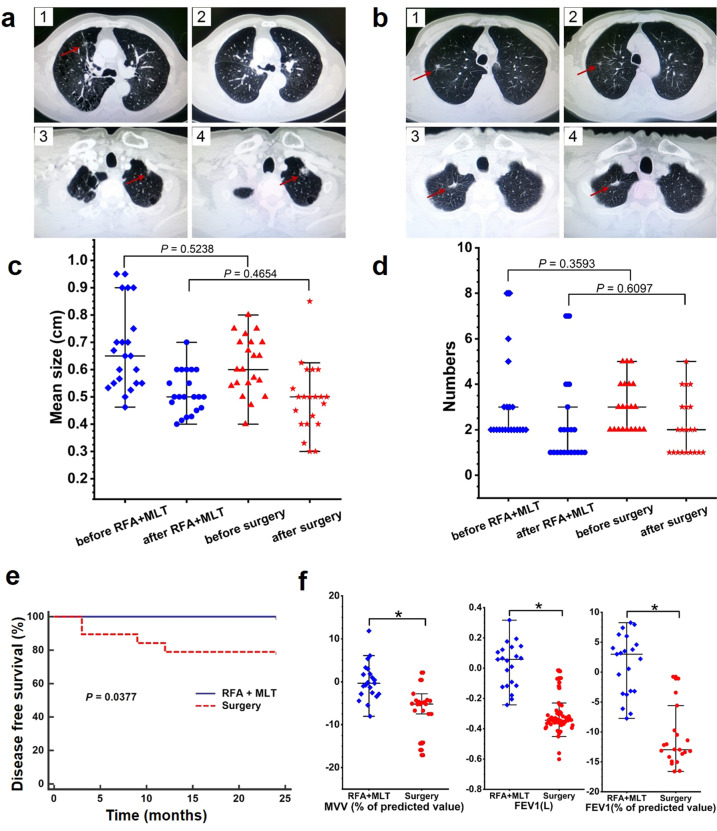
RFA + MLT treatment is significantly associated with clinical improvement. **a** Representative lung CT images for study participants with early lung tumor in the Surgery group with multiple lung nodules after 0 week (1), after 24 months (2), distal nodules after 0 week (3), and after 24 months (4). **b** Representative lung CT images for study participants with early lung tumor in the RFA + MLT group after 0 week (1), after 24 months (2), distal nodules after 0 week (3), and after 24 months (4) **c** The mean diameter of GGNs of patients before and after treatment (24 months) in the RFA + MLT and surgery groups (*P* < 0.05). **d** The numbers of GGNs of patients before and after treatment (24 months) in the RFA + MLT and surgery groups (*P* < 0.05). **e** Disease-free survival of patients in the RFA + MLT and surgery groups for 24 months via Logrank test (*P* = 0.0377). **f** The characteristics of pulmonary function of patients before and after treatment (12 months) in the RFA + MLT and surgery groups, including forced expiratory volume in 1 s (FEV_1_), FEV_1_ (% of predicted value), maximal ventilatory volume (MVV) (% of predicted value). (*P < 0.05)

As shown in Table 2, all patients in both groups were closely monitored after surgery. During the follow-up period of 2 years, the treated GNNs in all patients of both the RFA + MLT and Control groups disappeared or the sites became fibrous, suggesting 100% success rate for eliminating local GNNs. Especially for the RFA + MLT group, 18 cases (85.71%) developed fibrous deposits and 3 cases (14.29%) were decreased in size as described in Table 2 with typical CT diagnosis shown in Fig. 1a, b.

**Table 2:** Efficacy, complications, and cost of patients in the RFA+MLT and surgery groups

Parameters	RFA+MLT (*n* = 21)	Surgery (*n* = 21)	*P* value
CT images (*n*, %)			<0.0001
Fiber cord	18 (85.71)	0	
Decreased	3 (14.29)	0	
Disappeared	0	21	
Complications (*n*, %)			
Fever	2 (9.52)	5 (23.81)	0.2197
Chest pain	8 (38.10)	21 (100)	<0.0001
Hemoptysis	7 (33.33)	3 (14.29)	0.1522
Pneumothorax	6 (28.57)	13 (61.90)	0.0993
Pleural effusion	3 (14.29)	17 (80.95)	<0.0001
Pneumonia	0	4 (19.05)	0.0378
Delayed wound healing	0	1 (4.76)	0.3173
Length of stay in hospital (days)	3.32 (1.53)	14.25 (4.37)	<0.0001
Range (days)	2–7	8–26	—
In situ recurrence (*n*, %)			—
With	0	0	
None	21	21	
New lesions or nodule enlargement (*n*, %)			0.0377
With	0	4	
None	21	17	
Cost in hospital (RMB)	21,984 (3681)	66,114 (14,950)	<0.0001
Range (RMB)	14,303–32,662	39,119–91,413	—

During the 2-year follow-up, we detected significant improved clinical outcome of patients in the RFA + MLT group comparing to those in the Control group (Fig. 1a–f). Based on CT image diagnosis, there was no recurrence or any change in sizes of non-ablated nodules in 21 patients treated with RFA + MLT. However, for 21 patients with surgery only, 4 patients were found to have new or enlarged nodules in non-surgical areas, of which 2 patients were diagnosed as invasive adenocarcinoma cancer confirmed by further reoperation. As shown in Fig. 1e, 24 months later, the overall change of nodules in untreated areas was 0/21 vs 4/21 (recurrence rate: 0 vs 19%; *P* = 0.0377) for the RFA + MLT and Control groups, respectively. The recurrence rate of the Control group in our study was similar to what was recently observed in Asia.^7,8^ Also important, there was no impairment of lung function before and 12 months following this treatment in the RFA + MLT group, while a reduction of lung function was observed in the Control group (Fig. 1f and Supplementary Table S1). Consistently, more complications were detected in the Control group; these included chest pain in 21 individuals (100%) and pleural effusion in 17 cases (80.95%) (Supplementary Fig. S1). In addition, the hospital cost and length of stay for patients with RFA + MLT treatment were significantly decreased comparing to patients in the Control group (Supplementary Fig. S2). These data thus suggested that the combined treatment with RFA and MLT significantly inhibited the recurrence of lung nodules in non-ablated areas, with fewer pulmonary function injuries and complications.

### Combined treatment with RFA and MLT inhibits the growth of lung cancer in non-ablated areas in C57BL/6 mice

**Fig. 2:**
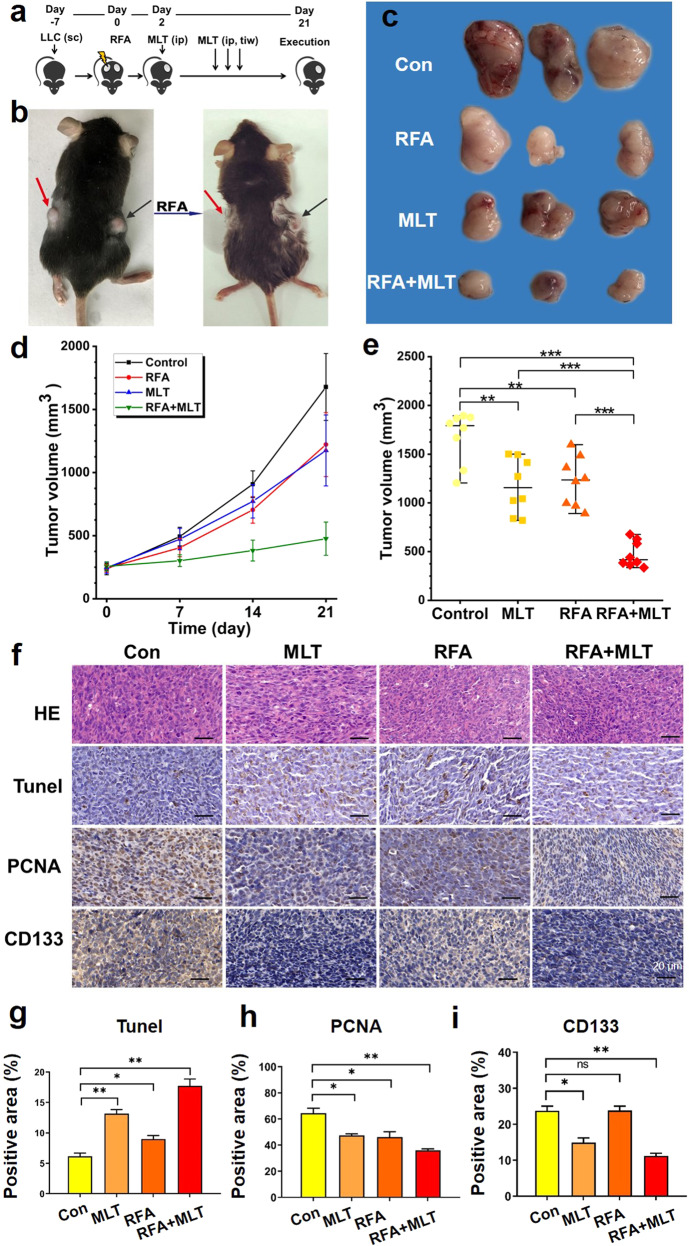
RFA + MLT treatment significantly inhibited tumor growth in non-ablated zone. **a** Experimental design of studies in C57BL/6 mice. Time in days relative to tumor treatment with radiofrequency ablation. **b** C57BL/6 mice were subcutaneously injected in bilateral flanks with Lewis lung cancer cells, and unilateral Lewis cancer cells were treated with radiofrequency ablation (red arrow for non-ablated zone in right and black arrow for ablated zone). **c** Images of tumor tissues after treatment with RFA, MLT, and RFA + MLT for 21 days. **d** Tumor volume after treatment with RFA, MLT, and RFA + MLT at different days. **e** Tumor volume after treatment with RFA, MLT, and RFA + MLT in 21 days. **f** Expression of HE, TUNEL, PCNA and CD133 in non-ablated tumor tissue samples (scale bar = 20 μm). The bar graph showed the level of **g** TUNEL, **h** PCNA, and **i** CD133 in non-ablated tumor tissue after treatment with MLT, RFA, and RFA + MLT, respectively. (*P < 0.05; **P < 0.01; ***P < 0.001)

To explore the mechanism underlying the inhibitory effect of combined treatment with RFA and MLT on tumor growth in non-ablated areas, we mimicked what was observed in patients with lung cancer mouse models. As shown in Fig. 2a, C57BL/6 mice were injected with mouse Lewis lung cancer cells bilaterally on the back; the animals were then randomly divided into four groups when the tumor reached about 250 mm^3^ (*n* = 8 for each group). These four groups are Control (mock treatment), RFA treatment, MLT treatment, and combined treatment with RFA and MLT (RFA + MLT), respectively. For tumors in the RFA and RFA + MLT groups, RFA exposure was performed to abolish tumors growing on one side thereby mimicking RFA-treated GGNs in early lung cancer patients while leaving tumors growing on contralateral side (Figs. 2b and Supplementary Fig S3). In terms of the non-ablated tumors, their growth was significantly inhibited after RFA + MLT treatment when compared to tumors in the Control group as well as in the RFA and MLT single treated groups (Fig. 2c, d, e). These data thus recapitulated what we observed in patients and demonstrated the synergistic effect of RFA and MLT on non-ablated tumor inhibition.

Consistent with what observed in tumor growth in our mouse model, immune histological staining revealed reduced cell proliferation (proliferating cell nuclear antigen staining) and increased cell death (terminal deoxynucleotidyl transferase-mediated dUTP-fluorescein nick end labeling staining) in tumors from RFA + MLT-treated mice compared to those from Control animals as well as single treated group (Fig. 2f, g, h). Importantly, we also detected a significant decreased expression of CD133 in tumor tissues from RFA + MLT animals, which suggested that fewer stem-like cancer cells survived after combined treatment with RFA and MLT (Fig. 2i).^21^ Of note, we did not detect any obvious changes in the body weight of the animals or abnormalities in the mice among the four groups (Supplementary Fig. S4), suggesting there was no significant alternation of nutritional status and toxicity with any treatments in this study.

### Combined treatment with RFA and MLT significantly increased NK cell infiltration into non-ablated tumor tissues and promoted NK antitumor immunity

**Fig. 3:**
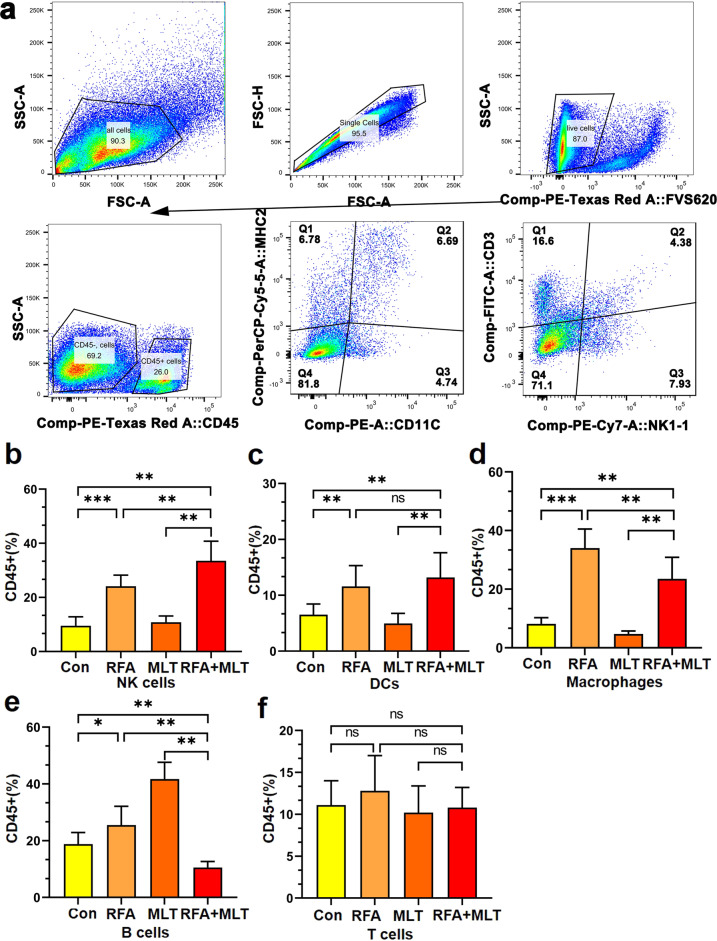
MLT promotes RFA-induced tumor infiltration of NK cells. **a** Flow cytometry showing NK cells from tumor tissue in C57BL/6 mice. Changes in survival of inflammatory cells of **b** NK cells, **c** DCs, **d** macrophages, **e** B cells, and **f** T cells from the non-ablated tumor tissues in the Control, RFA, MLT, and RFA + MLT groups, and the percentage of different lymphocytes were analyzed based on 10^4^ cells. (*P < 0.5, **P < 0.01; ***P < 0.001; ns, no significance)

It is known that RFA treatment could induce antitumor immunity, we thus checked whether the inhibited tumor growth in non-ablated areas resulted from an antitumor immune response initiated by RFA. Flow cytometry was used to access the presence of immune cells in tumor tissues among all groups. As shown in Figs. 3 and Supplementary Figs S5–S8, T cells, B cells, dendritic cells (DCs), and NK cells are all present in the lung tumor tissues. Although no significant differences for the infiltrated T and B immune cells were detected after RFA treatment, it significantly increased the infiltration of macrophages, DCs, and NK cells in tumors; MLT further promoted RFA-induced infiltration of NK cells but not macrophages and DCs in tumors (Fig. 3e, f, i). Furthermore, we performed flow cytometric experiments to detect some parameters (NK1-1, CD107a, CD69, tumor necrosis factor (TNF)-α, and interferon (IFN)-γ) on NK cells in tumors to investigated the state about tumor-infiltrating NK (TINK) activation, with the fresh tumor tissue from mouse. As shown in Supplementary Fig. S9, it was obvious that the CD45+/NK1.1+ cell-related parameters, including the activation makers (CD107a/CD69) and the effector molecules (TNF-α/IFN-γ), were more expressed in the RFA + MLT group. In addition, immunohistochemistry (IHC) staining was employed to trace some situations about antitumor functions of TINK cells in non-ablated tumor tissues. As shown in Supplementary Fig. S10, it was obvious that the antitumor functions were upregulated in RFA + MLT treatment compared to those from the Control group as well as single treated groups, such as the CD45+, the marker of NK cells (NKp46), the activation markers of TINK cell (CD69, CD107a), and even the effector molecules (TNF-α/IFN-γ). These findings thus suggested that the synergistic effect of RFA + MLT on non-ablated tumor growth may be mediated by NK antitumor immunity.

**Fig. 4:**
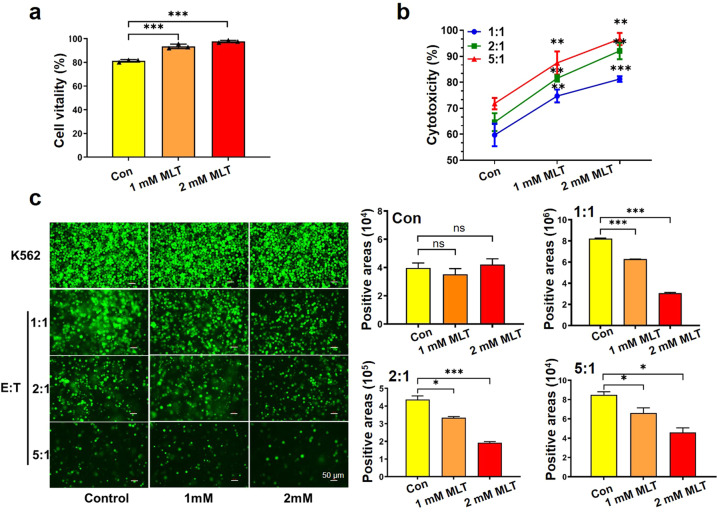
MLT promotes the viability and proliferation of NK cells and enhances the phagocytic ability of NK cells. **a** MLT promotes the viability and proliferation of NK cells. The cell vitality of NK92 cell after treatment with different concentrations of MLT for 24 h (0, 1, 2 mM) and the cell number was counted using Cellometer Auto T4 Cell Counter (*P < 0.05). **b** In vitro cytotoxicity of NK92 cells in co-culture with K562 cells at the indicated E:T ratios (1:1, 2:1, 5:1) with different concentrations MLT via CCK8 assay. **c** Representative fluorescence images and the bar graphs of MLT-treated NK cells in co-culture with K562 at different effector:target ratios for 24 h (scale bar = 50 μm). (*P < 0.05; **P < 0.01; ***P < 0.001)

Consistently, when we examined whether MLT directly influenced NK cell function, it was found that MLT dose-dependently enhanced viability of NK cells but not T cells in vitro (Figs. 4a, b and Supplementary Figs S11 and S12). Further, to investigate the functional consequences of NK cells, NK cells were co-cultured with K562 cells in the culture medium containing MLT or not. Increasing MLT concentrations efficiently promoted NK cell-mediated K562 cell killing (Figs. 4c and Supplementary Fig S11). These data thus suggested that, after combined treatment with RFA and MLT, RFA not only effectively eliminated the local tumor tissue but also stimulated NK-mediated antitumor immunity thereby inhibiting tumor growth in non-ablated areas.

### Combined treatment with RFA and MLT reduced tumor malignancy in non-ablated areas and altered tumor metabolism and the tumor microenvironment

**Fig. 5:**
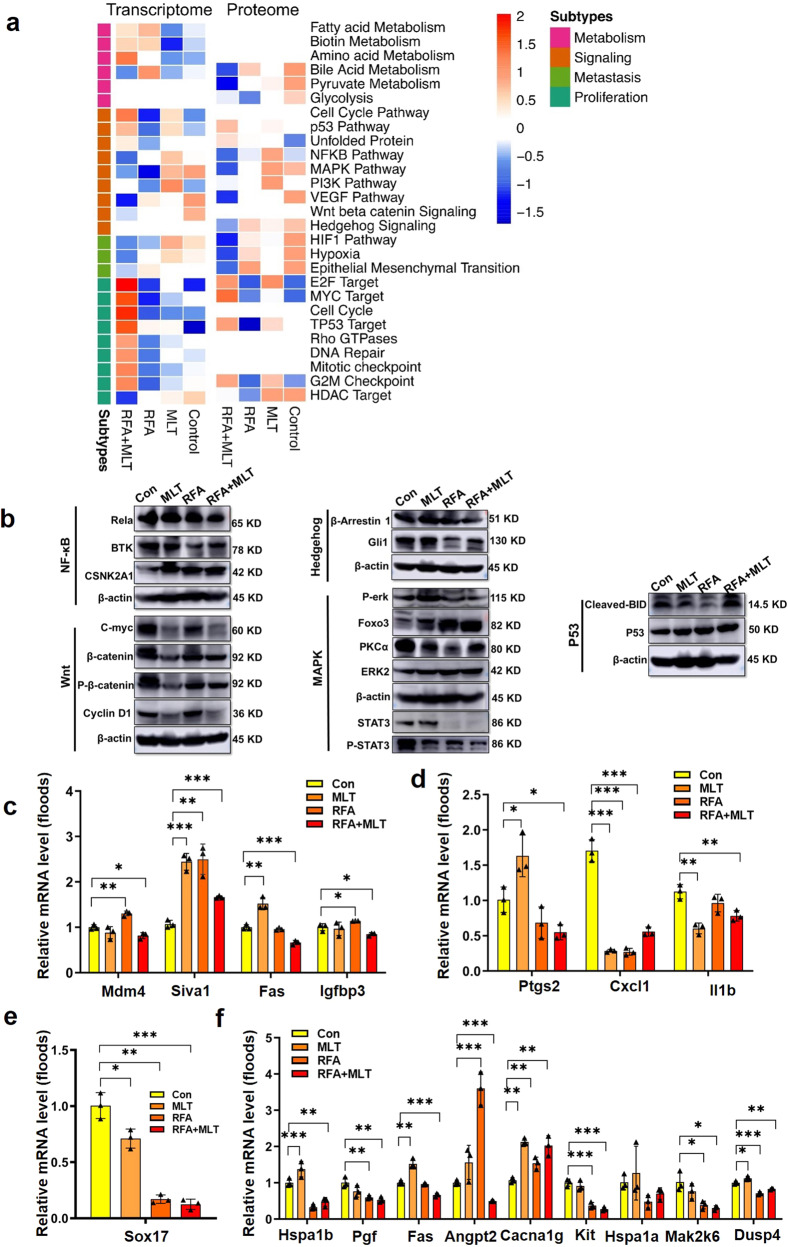
Combined treatment with RFA and MLT reduced tumor malignancy in non-ablated areas, altered tumor metabolism, and the tumor microenvironment. **a** The integrative multi-omics analysis of cancer proliferation and malignancy-related pathways. Heatmap of alteration pathways in the transcriptomic and proteomic subtypes. Left, alteration pathways identified from the transcriptome. Right, alteration pathways are identified from the proteome. Color of each cell represents the average ssGSEA enrichment scores of that subtype; red denotes activation and blue denotes inhibition. Blank cells represent non-enrichment. **b** The validation of cancer proliferation and malignancy-related pathways. Western blot analysis of P53 (Cleaved-BID and P53), Wnt (C-myc, β-catenin, P-β-catenin, and Cyclin D1), Hedgehog (β-Arrestin 1 and Gli1), MAPK (P-erk, Foxo3, PKC, ERK2, p-STAT3 and STAT3), and NFкB (Rela, BTK, CSNK2A1) pathway-altered proteins in the RFA + MLT, RFA, MLT, and Control groups; The normalized altered gene expression of **c** P53 (Mdm4, Siva1, Fas, and Igfbp3), **d** NFкB (Ptgs2, Cxcl1, and Il1b), **e** Wnt (Sox17), and **f** MAPK (Hspa1b, Pgf, Fas, Angpt2, Cacna1g, Kit, Hspa1a, Map2k6, Dusp4, and Il1b) signal pathways in the RFA + MLT, RFA, MLT, and Control groups (*P < 0.05; **P < 0 .01; ***P < 0. 001)

Combined treatment of RFA and MLT show remarkable inhibition on non-ablated tumors both in early lung patients and our lung cancer mouse models, we thus checked the remaining tumors treated with RFA and MLT by comprehensive analysis of transcriptomics and proteomics. As shown in Figs. 5a and Supplementary Figs. S13 and S14, we found a series of differential expression levels of genes/proteins for the non-ablated tumors in the RFA + MLT, RFA, and MLT vs Control group; these changes were mainly concentrated in 27 pathways. Especially for the RFA + MLT group, as shown in Fig. 5a, mitochondria-related pathways including oxidative phosphorylation (OXPHOS), fatty acid metabolism, cell cycle, MYC targets, E2F targets, DNA repair, and TP53 targets, etc., were distinctly upregulated. Consistently, the altered upregulated genes (Nduf family, Uqcr family, Cox family, etc.) were those mainly involved in pathways that are related to mitochondrial complexes, ribosomes, and key subunits in electron transport chains; we got similar results with proteomics analysis (Supplementary Figs. S15 and S16). These data thus implied that RFA + MLT treatment led to metabolic reprogramming of the cancer cells.

As shown in Fig. 5a, we further found that there were distinct changes in pathways related to cancer cell proliferation and apoptosis (mitogen-activated protein kinase (MAPK),^22^ P53,^23^) as well as in pathways related to cancer malignancy and stemness (nuclear factor кB (NFкB),^24,25^ Wnt,^26^ Hif1,^27^ and Hedgehog^28^). Western blotting and reverse transcriptase quantitative polymerase chain reaction (PCR) were performed to assess whether cancer proliferation and malignancy-related pathways (MAPK, NFкB, Wnt, Hif, Hedgehog pathways, and P53) were shifted to a lower malignancy state after RFA + MLT treatment. As shown in Fig. 5b, combined RFA + MLT treatment resulted in downregulation of Wnt (C-myc, P-β-catenin, and Cyclin D1), Hedgehog (β-Arrestin 1 and Gli1), MAPK (P-erk, Foxo3, PKC, ERK2, p-STAT3, and STAT3), and NFкB (Rela, BTK, CSNK2A1), as well as upregulation of P53 (Cleaved-BID and P53). These results are thus in line with what we observed in our mouse models (Fig. 2) that tumor growth and malignancy were inhibited after RFA + MLT treatment. In addition, the related genes in MAPK (Hspa1b, Pgf, Fas, Angpt2, etc.), NFкB (Ptgs2, Cxcl1, and Il1b), and Wnt (Sox17) signaling pathways were also downregulated. However, we also noticed the tumor-suppressive P53 pathway-related genes (Mdm4, Siva1, Fas, and Igfbp3) were upregulated in tumors from RFA + MLT-treated mice (Fig. 5c–f). Based on the multi-omics analysis, collectively, these data suggested that RFA + MLT treatment inhibited tumor growth and reduced tumor malignancy in line with our observation in patients, where RFA + MLT treatment significantly reduced tumor recurrence.

**Fig. 6:**
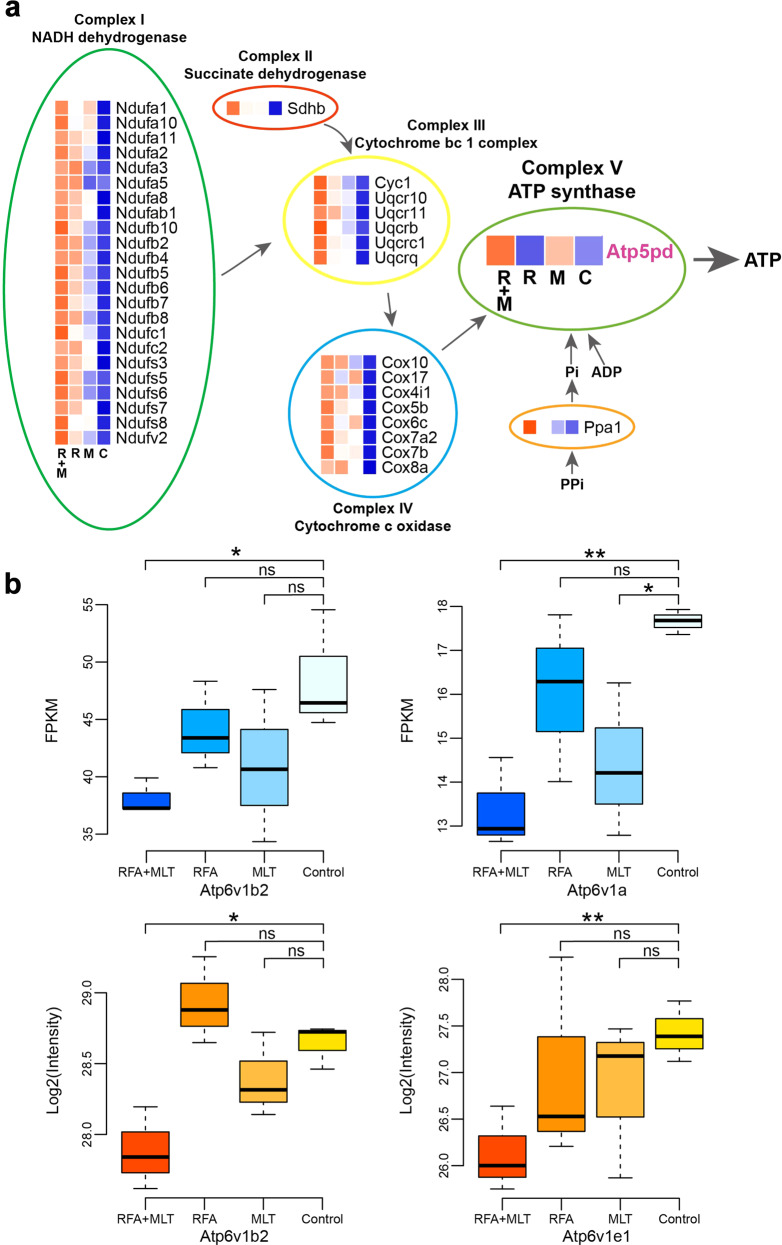
Multi-omics comprehensive analysis revealed genes and proteins that potentially correlate with mitochondrion in the RFA + MLT groups. **a** The integrative multi-omics data analysis of genes (black) and proteins (purple) related to Complex I, Complex II, Complex III, Complex IV, and Complex V in mitochondrion (FC > 1.2, *P* ≤ 0.05). **b** Boxplots representing the expression levels of V-ATPase-relevant genes (blue) and proteins (orange) in the RFA + MLT, RFA, MLT, and Control groups

As shown in Fig. 6a, comprehensive multi-omics analysis revealed upregulated expression of gene function in mitochondrial OXPHOS pathway Complexes I–IV (Nduf family, Sdhb, Cyc1, Uqcr family Cox family, Ppa1, and Atp5pd) after RFA + MLT treatment. Importantly, the distinct upregulated protein Atp5pd, which was closely related to Complex V, was more highly overexpressed in RFA + MLT-treated tumors than in the MLT only group. These data indicated the enhancement of OXPHOS and metabolic reprogramming in tumor cells with RFA + MLT treatment, that is, it converted Warburg-type metabolism to OXPHOS in the tumors. In addition, genes and proteins (Fig. 6b) (ATP6V1B2, ATP6V1A, ATP6V1E1), which were closely associated with a multi-subunit vacuolar H+-ATPase (V-ATPase) were also downregulated in tumors treated with RFA and MLT. It was known that V-ATPase played an important role in the acidity of the microenvironment in tumor tissues and was mainly conducted on cancer metastasis; this enzyme had visibly lower expression in tumor tissues treated with RFA + MLT, which further suggested reprogrammed tumor metabolism from Warburg-type metabolism to mitochondrial OXPHOS dependency.^29^

## Discussion

The postoperative recurrence of early lung cancer in patients with multi-GGNs after initial treatment is a big challenge worldwide.^30,31^ Surgery, the current standard treatment, efficiently removes the local tumor, but could not affect the malignant development of others nodules for early lung cancer patients with multi-GGNs.^32^ Commonly, patients with multiple GGNs faced multiple rounds of surgeries, which might result in serious pulmonary function injuries, disability, and even early death. Therefore, an innovative local combined system therapy to solve this problem is urgently needed. Herein we reported that RFA + MLT potently inhibited development of nodules in non-ablated areas for early lung cancer patients with multi-GGNs. The combined treatment of RFA and MLT not only addressed the shortcomings of nodular growth after RFA treatment alone but also overcame the limited efficacy of MLT alone in patients with a large tumor load; importantly, combined RFA + MLT caused minimal damage to normal lung tissue. The results in this current study indicated that combined treatment of RFA and MLT reduced the malignant transformation of non-ablated nodules and provided an innovative non-invasive method for clinical care of early lung cancer patients with multi-GGNs.

Compared to standard surgery, combined treatment of local (RFA) and systemic (MLT) for early lung cancer patients with multi-GGNs have several advantages, including side effect management and cost-saving. The combination of RFA and MLT not only wiped off local lung tumor burden via a minimally invasive ablation approach but also effectively reduced probability of multi-GGN enlargement and malignant transformation in non-ablated areas, which minimized lung function injuries and complications. Based on the follow-up data in our clinical study, it was clear that the tumor enlargement and malignant transformation after RFA + MLT treatment were greatly reduced comparing to the surgery group (0 vs 19%, *P* = 0.0377). The recurrence rate about standard care (surgery) in our study was consistent with the recurrence rate recently reported: 14.7–23.9%.^7,8,33^ The median follow-up period for all 285 patients was 27.6 months (range: 3.2–68.1) and there were 60 patients (21.1%) who experienced recurrence or distant metastases in Peking University People’s Hospital.^7^ Fudan University Cancer Hospital reported that recurrence for 65 patients (14.7%) in 442 patients with multi-GGNs in a median follow-up of 21 months.^8^ Fabian et al. reported 16 (23.9%) cases of recurrent nodules in 67 patients with multiple primary lung cancers, after an average follow-up of 45.5 months.^33^ Thus, compared with the current first-line treatment with surgery, RFA + MLT treatment could efficiently inhibit multi-GGN development in non-ablated lung areas resulting in greatly improved clinical outcome.

As demonstrated in our mouse lung tumor model, the combined treatment of RFA and MLT effectively inhibited tumor growth in non-ablated areas, which may result from the enhanced NK antitumor immunity. Meanwhile, it was obvious the downregulation of tumor stem-related pathways, along with the suppression of aerobic glycolysis and the enhancement of OXPHOS after RFA + MLT treatment. Reprogramming cancer cell metabolism from aerobic glycolysis to OXPHOS is reported as a proposed mechanism for anticancer activity of MLT.^34^

Data from our mouse tumor model, fully consistent with our clinical findings, was suitable for a detailed mechanistic study. It was reported that RFA not only destroyed local tumor tissues but also induced antitumor immunity.^10,35^ Consistently, our results show that RFA significantly increased the infiltration of immune cells including macrophages, DCs and NK cells in non-ablated tumor tissues, and this became more obvious after RFA + MLT treatment. NK cells showed spontaneous cytotoxic activity against tumor cells and released cytokines, which modulate the function of other immune cells, and thus played a key role in tumor immunotherapy.^36–38^ In our study, we found that RFA promotes NK cell infiltration in non-ablated tumor tissue and the percentage of macrophages and DCs was also significantly increased after RFA + MLT treatment. It was supposed that macrophages and DCs also played a part in NK anticancer immunity for RFA + MLT treatment. This NK cell infiltration was further enhanced in the RFA + MLT group consistent with synergistic effect of RFA and MLT on tumor growth in our mouse model. Our in vitro data also showed that exogenous MLT treatment took a positive effect on NK cell viability,^39^ and it further strengthened NK cell killing of target cells in a dose-dependent manner. These data thus suggested a model that MLT enhances RFA-induced NK antitumor immunity to significantly limit tumor growth in non-ablated areas. And it was reported that MLT could also potentiate the proliferation and activities of NK cells via triggering the release of interleukin (IL)-2.^40^ Here the enhancement of NK anticancer immunity in RFA + MLT treatment maybe closely related to the increased production of cytokines.

Our clinic data showed that combined treatment of RFA + MLT greatly reduced early lung cancer recurrence, which could be explained via our multi-omics analysis with tumor samples in the Control and RFA + MLT groups.^41,42^ We observed that the P53 pathway was upregulated while the MAPK, NFкB, Wnt, and Hedgehog pathways were downregulated after RFA + MLT treatment. It is well known that P53 functions as a tumor suppressor and monitors tumor progress, and a gain of function of P53 pathway suggests a reduced malign state of treated tumors. MAPK plays a vital role in cancer cell apoptosis and proliferation.^22,23,43^ As for NFкB, it is a major factor in cancer progression because it influences proliferation, metastasis, metabolic reprogramming, etc.^24,25^ Hypoxia-inducible factor-1α normally cooperates with NFкB to maintain the malignancy of tumors.^27^ Of importance, Wnt is a highly critical carcinogenic pathway related to immune escape, while Hedgehog regulates the vitality of tumor stem cells to maintain tumor malignancy.^26,28^ We also detected the upregulation of MAPK, NFкB, Wnt, and Hedgehog pathways. These pathways were frequently activated in multiple types of tumors, and their suppression often led to reducing cancer cell growth and compromised stem state of tumors. All these data thus suggested that combined treatment of RFA and MLT not only inhibited the growth of tumors but also constrained their “cancer state,” which was in line with our clinical findings.

Consistently, we found a strong metabolic reprogramming of cancer cells from their typical aerobic glycolysis state to mitochondrial OXPHOS state in RFA + MLT treatment. Recently, it was reported that the antitumor immunity of NK was closely related to the mitochondria and tumor microenvironment.^44^ The combined treatment of RFA and MLT likely improved the microenvironment of tumor mitochondria, which may be related to the enhancement of NK antitumor immunity in the non-ablated area (Supplementary Fig. S17). Also, V-ATPase showed visibly lower expression in tumor tissues in RFA + MLT, which suggested that ATPase expression was inhibited and the acidic microenvironment of tumor tissues was lowered. Tumorigenesis is a complex process that involves multi-signal pathways.^43^ And RFA + MLT treatment caused upregulated expression of lipid metabolism, DNA repair pathways, cell cycle, and mitotic checkpoints (G2M, E2F target), which suggest that the tumors were adjusted to a lower malignancy.^45,46^

In conclusion, we established an innovative treatment combination (RFA + MLT) that resulted in marked clinical improvement and caused minimal lung function damage and even associated complications. Most importantly, this unique treatment inhibited multiple pulmonary nodule development in non-ablated lung regions. Clinically, these findings indicated obvious treatment advantages to minimize trauma and reduce tumor recurrence in patients. Thus, the combination with RFA and MLT could provide an effective clinic solution for early lung cancer with multi-GGNs to avoid surgery and fill the gap in this field with an innovative strategy for integration of treatment and prevention. This novel combination of local and systemic treatment could have a profound impact on early lung cancer care.

## Materials and methods

### Clinical investigations and data analysis

The study was approved by the ethics committee and was funded by Shanghai Tenth People’s Hospital Affiliated with Tongji University. This study followed Consolidated Standards of Reporting Trials guidelines. Our primary clinical objective was to assess the improvement of RFA + MLT treatment in patients of early lung cancer with multiple nodules and to examine potential mechanisms involved in mitochondria. The study was a non-randomized and open label, and all participants or their guardians provided informed consent before they participated in the study.

For this study, eligible patients aged >18 years had biopsy-proven histological diagnosis of AAH, AIS, or adenocarcinomas without mediastinal lymph node enlargement; patients had no history of previous surgical treatment, radiotherapy, chemotherapy, or local treatment. CT guidance was used to treat tumors under local anesthesia. All patients were treated with the same RFA electrodes (Celon proSurge: T 20) measuring 2 cm in diameter and at least 10 mm larger than the diameter of the target tumor. Multiple overlapping ablations were performed, when needed, in different parts of the tumor in order to cover the entire volume. Patients were prescribed to orally take MLT (5 mg/day) for 12 months 1 week after RFA treatment. After that, thin layer of low-dose CT was used to assess local efficacy in patients with RFA + MLT or surgery at 1, 3, 6, 9, and 12 months, while a whole body and laboratory examinations including tumor marker detection and biochemical examination were also performed. And the recurrence probability of patients was drawn by Kaplan–Meier method.

### Mouse studies

Male and female C57BL/6 mice were purchased from Shanghai SLAC Laboratory Animal Co. Ltd and kept under specified pathogen-free conditions in the animal house of Shanghai Tongji University north campus. All of the experiments were performed according to the guidelines for animal care of the Tongji University Animal Experiments Committee. For follow-up animal experiments, 5-week-old mice were used and randomly divided into four groups (*n* = 8 for each group), labeling as the Control group, RFA group, MLT group, and RFA + MLT group, respectively.

Lewis lung cancer cells were purchased from Shanghai Zhong Qiao Xin Zhou Biotechnology Co., Ltd. and its final concentration was adjusted to 10^6^ cells/mL with phosphate-buffered saline (PBS) buffers as used previously. The C57BL/6 model was injected with 10^6^ total colony-forming units/ear of Lewis lung cancer cells on the bilateral side back for subcutaneously tumorigenesis previously. Evaluation of tumor size was performed every 2 days using calipers. Tumor volumes were scored with the formula (*A* & *B*^2^) & 0.4, in which *A* is the largest and *B* is the shortest dimension. The mice were selected for serial experiments when their volumes measured about 250 mm^3^ at 8 days. For the RFA and RFA + MLT groups, mice were anesthetized by chloral hydrate solution (35 mg/mL) injection and the tumor area was disinfected with alcohol. An RFA needle with active tip of 8 mm (RFA system, Beijing Welfare Electronics Co.) was inserted and placed in the middle of the tumor, which were on the right side. Treatment then was started by delivering RFA energy, and the parameters in RFA treatment were 60 W, 70 °C, which lasted for 3 min. For the MLT and RFA + MLT groups, MLT solution (10 mg/kg, 0.1 mL) was intraperitoneally injected on the same day as with RFA treatment. The tumor volume of each mouse was measured every 7 days in the RFA + MLT, RFA, MLT, and Control groups. All mice were sacrificed to collect the subcutaneous tumor tissues on the left side until 3 weeks after RFA treatment. The tumors were removed, paraffin embedded, fixed in 4% paraformaldehyde for 24 h, and sliced into 5-μm-thick sections for IHC analysis and following transcriptomic and proteomics assay.

### NK92 cell assay

NK92 cells were cultured complete RPMI 1640 medium containing IL-2 (200 U/mL). K562 cells were also transduced with pMGIR-GFP-mCherry vector. Transduced cells were selected for their expression of GFP or mCherry with a FACSAria (BD Biosciences) cell sorter. The K562 cells were reseeded in a 12-well flat bottom plat at a density of 1.5 × 10^5^, and then NK92 cells were harvested and added at the indicated E:T ratios and co-cultured for 24 h in the presence of different concentrations MLT in 2 mL complete medium (RPMI 1640 medium with 10% fetal bovine serum). Finally, the stained cells were imaged under an inverted Nikon Ti-S fluorescence microscope at scale bar = 50 μm.

Cell viability was determined using a CCK-8 Kit (Yeasen, Shanghai, China). Briefly, 1.5 × 10^5^ K562 cells/well were seeded into 12-well plates treated with different concentrations of MLT, and then NK92 cells were harvested and added at the indicated E:T ratios and co-cultured for 24 h, followed by addition of CCK-8 to each well at 24 h. Optical density (OD) was measured at 450 nm after 3-h incubation in the 37 °C incubator. The cytotoxicity was calculated as follows:

$$\% {\rm{Cytotoxicity}} = \left[ 1 - \frac{{\rm{OD}}\left({\rm{effector}} + {\rm{target}}\;{\rm{cells}} \right) - {\rm{OD}}({\rm{effector}}\;{\rm{cells}})}{{{\rm{OD}}({\rm{target}}\;{\rm{cells}})}} \right] \times 100\%$$

Cytolytic assay of NK92 cells and K562 cells was performed using carboxyfluorescein succinimidyl ester (CFSE)–7-aminoactinomycin D (7AAD) via flow cytometry analysis. Briefly, 2 × 10^5^ NK92 cells/well were seeded into 6-well plates treated with different concentrations of MLT for 48 h, and then NK92 cells were harvested and added at the indicated E:T ratios and co-cultured for 4–5 h; inside, the K562 cells was labeled by CFSE. Finally, each sample was washed with cell staining buffer twice and then was assessed using 7AAD. The results were analyzed with a BD FACSCanto II flow cytometry system (BD Biosciences), and the percentage of apoptotic cells was analyzed based on 10^4^ cells.

### Flow cytometry

Four weeks after RFA treatment, tumor-infiltrating lymphocytes were harvested from the mice on the sacrifice day. After washing the tissue with PBS, it was dissected into smaller fragments using a scalpel, and tumor tissues of equal weight in different groups were chosen for the measures of different lymphocytes. Next, 3 mL of enzymatic hydrolysate medium (supplemented with collagenase I (0.2 %), DNase (0.002 %), and Dulbecco’s modified Eagle’s medium) was added, and the tumor was disintegrated by rubbing against the mesh. Then it was diluted with 5 eq enzymatic hydrolysate medium and followed by being homogenized for 20 min and rested horizontally for 5 min. The mix was centrifuged for 5 min at 1000 × *g* at 4 °C. The cells suspension was then filtered and treated with FC block and ammonium-chloride-potassium (ACK) lysing buffer (Quality Biological, USA) before cellular staining. For the evaluation of cytotoxic CD8 T lymphocytes (CTL), the suspended cells were incubated for 30 min at 4 °C with the FITC-conjugated anti-mouse CD antibody (T cells:CD45+/CD3+/NK1.1−, NK cells: CD45+/CD3−/NK1.1+, DCs: CD45+/CD11C+/MHC2+; B cells: CD45+/CD45R+/CD3−, Macrophages: CD45+/F4-80+/CD11B+), and the parameter antibodies (CD 69: CD69-PE-CY7; CD107a: CD107a-APC; TNF-α, BV605; IFN-γ, BV480) about NK cells in tumor tissue were got from BD. Finally, each sample was washed with cell staining buffer twice and then was assessed using a three-color FACSCalibur flow cytometer (BD, Biosciences, CA, USA). The results were analyzed with a BD FACSCanto II flow cytometry system (BD Biosciences), and the percentage of different lymphocytes was analyzed based on 10^4^ cells.

### Western blot analysis

Tumor tissue were incubated to study the NFкB, Wnt, P53, Hedgehog, and MAPK pathways. Freshly tumor tissues were lysed in radioimmunoprecipitation assay (RIPA) buffer (150 mM sodium chloride (NaCl), 1% Triton X-100, 0.5% deoxycholate, 0.1% sodium dodecyl sulfate (SDS), and 50 mM Tris(hydroxymethyl). Concisely, lysis buffer was added to resuspend the cell pellet, containing the protease/phosphatase inhibitor and phenylmethanesulfonylfluoride (PMSF). The cellular suspensions were vortexed for 15 s, and then incubation on ice was done for 10 min. Following that, the cell lysate was centrifuged for 10 min at 12,000 rpm and 4 °C. Then 50 μg of protein extract was separated on SDS-polyacrylamide gel electrophoresis with 5% stacking and 10% running gel. Blotting onto the membrane containing polyvinylidene difluoride (PVDF; Roche Diagnostics) was done by the semidry immunoblotting. These membranes blockade was done via treatment with 0.5% Tween 20 in PBS for 2 h under shaking conditions at 25 °C. After that, the membranes were treated with monoclonal antibodies against Cleaved-BID (2003, CST), P53 (48818, CST), BTK (56044, CST), C-myc (18583, CST), β-catenin (8480, CST), P-β-catenin (4176, CST), Cyclin D1 (55506, CST), β-Arrestin 1 (12697, CST), Gli1 (2553, CST), P-erk (3371, CST), Foxo3 (2497, CST), PKCα (2056, CST), ERK2 (9108,CST), STAT3 (12640, CST), p-STAT3 (9145, CST) Rela (8214, CST), BTK (56044, CST), CSNK2A1 (2656, CST), and an internal control protein (β-actin) overnight at 4 °C (1:1000; Santa Cruz Biotechnology). Then, after washing the PVDF membranes, the incubation of the membranes with rabbit anti-goat secondary antibody for the above-mentioned proteins and rabbit anti-mice antibody mixed with horseradish peroxidase (1:5000; diluted in PBS) was performed for 1 h at 25 °C on the shaker. The protein bands were imagined via the Electrochemiluminescence Kit (Roche Diagnostics) and western blotting imaging instrument (Amersham Imager 600).

### Real-time quantitative reverse transcription PCR

TRIZOL (T9424, Sigma) was used to extract mRNA. About 100 mg of tumor tissues were ground and homogenized in 1 mL of TRIZOL Lysis Reagent. Afterwards, the homogeneous product was centrifuged for 10 min at 12,000 × *g* and 4 °C; the supernatant was removed and transferred to a new microtube. In the next step, 200 μL of chloroform was added to the separated supernatant and vigorously stirred for 15 s. Then, microtubes were re-centrifuged for 15 min at 12,000 × *g* and 4 °C. The aqueous phase was removed and 600 μL of isopropyl alcohol was added and centrifuged at 12,000 × *g* to extract total RNA. Synthesis of cDNA was carried out using the Takara’s cDNA Synthesis Kit, according to the manufacturer’s instructions. Expression of the desired genes was measured using real-time PCR and the results were quantified using the 2^−ΔΔCT^ formula.^47^ PCR reactions were performed using QuantStudio Dx. Forty cycles were considered for each cycle of real-time PCR. And the temperatures of each cycle were set at 94 °C for 20 s, 60–58 °C for 30 s, and 72 °C for 30 s. GAPDH was used as the reference gene to measure relative gene expression and melting curve analysis was performed to control the specificity of the product. The sequence of the primers is reported in Supplementary Table S2.

### Transcriptome analysis

Transcriptome analysis was performed on three biological replicates for the RFA + MLT, RFA, MLT, and Control groups using mRNA sequencing (mRNA-seq). Total RNA was extracted from the tissues using Trizol (Invitrogen, Carlsbad, CA, USA) according to the instructions of the manual. About 60 mg of tissue were ground into powder by liquid nitrogen in a 2 mL tube, followed by being homogenized for 2 min and rested horizontally for 5 min. The mix was centrifuged for 5 min at 12,000 × *g* at 4 °C, then the supernatant was transferred into a new EP tube with 0.3 mL chloroform/isoamyl alcohol (24:1). The mix was shacked vigorously for 15 s, and then centrifuged at 12,000 × *g* for 10 min at 4 °C. After centrifugation, the upper aqueous phase where RNA remained was transferred into a new tube with equal volume of supernatant of isopropyl alcohol, then centrifuged at 13,600 rpm for 20 min at 4 °C. After deserting the supernatant, the RNA pellet was washed twice with 1 mL 75% ethanol, then the mix was centrifuged at 13,600 rpm for 3 min at 4 °C to collect residual ethanol, followed by the pellet air dry for 5–10 min in the biosafety cabinet. Finally, 25–100 µL of DEPC-treated water was added to dissolve the RNA. Subsequently, total RNA was qualified and quantified using a Nano Drop and Agilent 2100 bioanalyzer (Thermo Fisher Scientific, MA, USA). Oligo(dT)-attached magnetic beads were used to get purified mRNA. Purified mRNA was fragmented into small pieces with fragment buffer at appropriate temperature. Then first-strand cDNA was generated using random hexamer-primed reverse transcription, followed by a second-strand cDNA synthesis. Afterwards, A-Tailing Mix and RNA Index Adapters were added by incubating the end repair. The cDNA fragments obtained from previous step were amplified by PCR, and products were purified by Ampure XP Beads, then dissolved in EB solution. The product was validated on the Agilent Technologies 2100 bioanalyzer for quality control. The double-stranded PCR products from previous step were heated denatured and circularized by the splint oligo sequence to get the final library.

The sequencing RNA data was filtered with SOAP nuke (v1.5.2) by (1) removing reads containing sequencing adapter, (2) removing reads whose low-quality base ratio (base quality ≤5% is >20%; (3) removing reads whose unknown base (“*N*” base) ratio is >5%; afterwards clean reads were obtained and stored in FASTQ format. The clean reads were mapped to the reference genome using HISAT2 (v2.0.4). Bowtie2 (v2.2.5) was applied to align the clean reads to the reference coding gene set, then expression level of gene was calculated by RSEM (v1.2.12). The heatmap was drawn by pheatmap (v1.0.8) according to the gene expression in different samples. Essentially, differential expression analysis was performed using the DESeq2 (v1.4.5) with *Q* value ≤0.05.

Differentially expressed gene (DEG) analyses between the RFA + MLT, RFA, MLT, and Control groups were performed as follows: to get insight into the change of phenotype, Gene Ontology and Kyoto Encyclopedia of Genes and Genomes (KEGG) enrichment analysis of annotated DEG was performed by Phyper based on hypergeometric test. The significant levels of terms and pathways were corrected by *Q* value with a rigorous threshold (*Q* value ≤0.05) by Bonferroni. To detect the DEGs at the RNA level, genes detected (Fragments Per Kilobase of transcript per Million mapped reads (FPKM) > 1) in at least six samples were considered, including the RFA + MLT, RFA, MLT, and Control groups. A pseudo FPKM value of 0.0001 was added to each empty element before the statistical test. Wilcoxon rank-sum test (as implemented in R software) was used to assess the DEGs. Genes with *P* < 0.05 and fold change ≥1.5 were considered to be DEGs. KEGG enrichment analysis was performed with “clusterProfiler” package in R. *P* ≤ 0.05 was considered statistically significant.

### Proteomic analysis

For the RFA + MLT, RFA, MLT and Control groups, proteins from three biological replicates of each sample were extracted. Briefly, 100 mg tumor tissues were put into the mixture of RIPA and 1% protease inhibitor (PMSF) and then were rapidly ground via an automatic sample rapid grinding instrument. Next, the liquid supernatant in tissue debris were collected by centrifugation at 15,000 rpm for 15 min at 4 °C, and these samples was preserved at −80 °C for 48 h. In succession, these thawing samples were purified by centrifugation at 12,000 rpm for 10 min at 4 °C twice. Finally, these depurated protein samples were prepared for liquid chromatography–tandem mass spectrometry (LC-MS/MS) analysis via basic bovine serum albumin replication, precipitation, digestion, and desalting.

LC-MS/MS analysis was performed using a maXis impact mass spectrometer (Bruker, Bremen, Germany). Injections consisted of 10 μL tryptic peptides onto a Dionex Trap column (100 μm × 2 cm × 5 μm), and samples’ separation were performed on a C18 RP column (75 μm × 15 cm × 3 μm). The flow rate was 300nL/min and the linear gradient was over 90 min (from 2 to 35% phase B; mobile phase A consisted of H_2_O in 0.1% formic acid (FA) and B consisted of ACN in 0.1% FA). Full MS spectra were acquired on Bruker Maxis Impact UHR Q-TOF. Full mass scan ranging from 350 to 1500 *m*/*z* with a mass resolution of 70,000 and the 10 most abundant MS ions were chosen for collision-induced dissociation fragmentation, and the corresponding data were acquired with the Maxquant software (Swiss-Prot database (Taxonomy: *Homo sapiens*, 20,205 entries)).

For bioinformatics pathway analysis, detection of proteins upregulated or downregulated in the RFA + MLT, RFA, MLT and Control groups were performed as follows:

To detect the differentially expressed proteins, proteins detected (intensity >0) in at least 2 samples were considered, including the RFA + MLT, RFA, MLT, and Control groups. A pseudo intensity value of 2.4871 was added to each empty element before the statistical test. Wilcoxon rank-sum test (as implemented in R software) was used to assess the differentially expressed proteins. Proteins with P < 0.05 and fold change ≥1.5 were considered to be DEGs.

### Functional enrichment analysis of signature genes and proteins

To gain further insight into biological implications, we performed gene set variation analysis (GSVA) to identify the pathway alterations that underlie our different experiment groups, using the R/Bioconductor package GSVA. GSVA analysis requires the following two main input arguments: the gene expression data and a collection of gene sets. In this study, the gene sets were obtained from the MSigDB database (http://software.broadinstitute.org/gsea/msigdb/index.jsp). Another input was the expression matrix of the signature genes or proteins. For protein signatures, the gene identifiers were identified by the UniProt ID and mapped to the Human Genome Nomenclature Committee’s HUGO symbol (http://www.genenames.org/).

Through knowledge-based annotation and overlapping genes, we refined identified pathways to the core functional pathways related to the following five biological function categories: cell proliferation, immune response, metastasis, metabolism, and signaling pathways.

### Statistical analysis

All statistical analyses were performed using the MedCalc Software (Version 19.5.6, MedCalc Software bvba, Ostend, Belgium) and figures were generated using the GraphPad and Origin softwares. Continuous data are presented as mean ± SEM, categorical variables as number (percentages). To determine statistical significance, unpaired *t* test, Mann–Whitney *U* test, Chi-square test, or one-way analysis of variance with multiple-comparison corrections were performed. Kaplan–Meier survival curve and log-rank test were used to estimate the disease-free survival of the patients in different groups. A *P* value of <0.05 was considered statistically significant.

## Supplementary information

supplemental material

## Data Availability

The data sets used for the current study are available from the corresponding author upon reasonable request.

## References

[1] Liu X (2020). The mortality of lung cancer attributable to smoking among adults in China and the United States during 1990-2017. Cancer Commun..

[2] Yang D, Liu Y, Bai C, Wang X, Powell CA (2020). Epidemiology of lung cancer and lung cancer screening programs in China and the United States. Cancer Lett..

[3] Horeweg N (2014). Lung cancer probability in patients with CT-detected pulmonary nodules: a prespecified analysis of data from the NELSON trial of low-dose CT screening. Lancet Oncol..

[4] Robbins HA, Berg CD, Cheung LC, Chaturvedi AK, Katki HA (2019). Identification of candidates for longer lung cancer screening intervals following a negative low-dose computed tomography result. J. Natl Cancer Inst..

[5] Hirsch FR (2017). Lung cancer: current therapies and new targeted treatments. Lancet.

[6] Wang L-Y, Cui J-J, Guo A-X, Yin J-Y (2018). Clinical efficacy and safety of afatinib in the treatment of non-small-cell lung cancer in Chinese patients. Onco. Targets Ther..

[7] Zhang Z (2016). Surgical outcomes of synchronous multiple primary non-Small cell lung cancers. Sci. Rep..

[8] Zhang Y (2020). Imaging features suggestive of multiple primary lung adenocarcinomas. Ann. Surg. Oncol..

[9] Picchi SG (2020). RFA of primary and metastatic lung tumors: long-term results. Med. Oncol..

[10] Qi X (2020). Synergizing sunitinib and radiofrequency ablation to treat hepatocellular cancer by triggering the antitumor immune response. J. Immunother. Cancer.

[11] Kroeze, S. G. C. et al. Stereotactic radiotherapy combined with immune- or targeted therapy for metastatic renal cell carcinoma. *BJU Int*. 10.1111/bju.15284 (2020). 10.1111/bju.15284 33113260

[12] Uhlig J (2019). Comparison of survival rates after a combination of local treatment and systemic therapy vs systemic therapy alone for treatment of stage IV non-small cell lung cancer. JAMA Netw. Open.

[13] Chen DW (2020). SHP-2 and PD-L1 inhibition combined with radiotherapy enhances systemic antitumor effects in an anti-PD-1-resistant model of non-small-cell lung cancer. Cancer Immunol. Res..

[14] Stein RM (2020). Virtual discovery of melatonin receptor ligands to modulate circadian rhythms. Nature.

[15] Mauriz JL, Collado PS, Veneroso C, Reiter RJ, González-Gallego J (2013). A review of the molecular aspects of melatonin’s anti-inflammatory actions: recent insights and new perspectives. J. Pineal Res..

[16] Manchester LC (2015). Melatonin: an ancient molecule that makes oxygen metabolically tolerable. J. Pineal Res..

[17] Gil-Martín E, Egea J, Reiter RJ, Romero A (2019). The emergence of melatonin in oncology: focus on colorectal cancer. Med. Res. Rev..

[18] Zhang W-x, He B-m, Wu Y, Qiao J-f, Peng Z-y (2019). Melatonin protects against sepsis-induced cardiac dysfunction by regulating apoptosis and autophagy via activation of SIRT1 in mice. Life Sci..

[19] Reiter RJ (2017). Melatonin, a full service anti-cancer agent: inhibition of initiation, progression and metastasis. Int. J. Mol. Sci..

[20] Reiter RJ, Sharma R, Ma Q, Rorsales‑Corral S, Chuffa LG (2020). Melatonin inhibits Warburg‑dependent cancer by redirecting glucose oxidation to the mitochondria: a mechanistic hypothesis. Cell. Mol. Life Sci..

[21] Barzegar Behrooz A, Syahir A, Ahmad S (2019). CD133: beyond a cancer stem cell biomarker. J. Drug Target..

[22] Reddy D, Kumavath R, Tan TZ, Ampasala DR, Kumar AP (2020). Peruvoside targets apoptosis and autophagy through MAPK Wnt/β-catenin and PI3K/AKT/mTOR signaling pathways in human cancers. Life Sci..

[23] Jiang L (2015). Ferroptosis as a p53-mediated activity during tumour suppression. Nature.

[24] Dimitrakopoulos F-ID, Kottorou AE, Kalofonou M, Kalofonos HP (2020). The fire within: NF-κB involvement in non-small cell lung cancer. Cancer Res..

[25] Friedmann-Morvinski D (2016). Targeting NF-κB in glioblastoma: a therapeutic approach. Sci. Adv..

[26] Zhan T (2019). MEK inhibitors activate Wnt signalling and induce stem cell plasticity in colorectal cancer. Nat. Commun..

[27] Shin D (2020). Midkine is a potential therapeutic target of tumorigenesis, angiogenesis, and metastasis in non-small cell lung cancer. Cancers.

[28] Szczepny A (2017). The role of canonical and non-canonical Hedgehog signaling in tumor progression in a mouse model of small cell lung cancer. Oncogene.

[29] Wang P (2017). Expression and transcriptional regulation of human ATP6V1A gene in gastric cancers. Sci. Rep..

[30] Robles AI, Harris CC (2017). Integration of multiple “OMIC” biomarkers: a precision medicine strategy for lung cancer. Lung Cancer.

[31] Chang JY (2015). Stereotactic ablative radiotherapy versus lobectomy for operable stage I non-small-cell lung cancer: a pooled analysis of two randomised trials. Lancet Oncol..

[32] Farago AF, Keane FK (2018). Current standards for clinical management of small cell lung cancer. Transl. Lung Cancer Res..

[33] Fabian T, Bryant AS, Mouhlas AL, Federico JA, Cerfolio RJ (2011). Survival after resection of synchronous non–small cell lung cancer. J. Thorac. Cardiovasc. Surg..

[34] Reiter RJ, Sharma R, Rosales-Corral S (2021). Anti-warburg effect of melatonin: a proposed mechanism to explain its inhibition of multiple diseases. Int. J. Mol. Sci..

[35] Palussiere J (2018). Radiofrequency ablation of stage IA non-small cell lung cancer in patients ineligible for surgery: results of a prospective multicenter phase II trial. J. Cardiothorac. Surg..

[36] Au KM, Park SI, Wang AZ (2020). Trispecific natural killer cell nanoengagers for targeted chemoimmunotherapy. Sci. Adv..

[37] Chiossone L, Dumas P-Y, Vienne M, Vivier E (2018). Natural killer cells and other innate lymphoid cells in cancer. Nat. Rev. Immunol..

[38] Paul S, Lal G (2017). The molecular mechanism of natural killer cells function and its importance in cancer immunotherapy. Front. Immunol..

[39] Carrillo-Vico A, Lardone PJ, Alvarez-Sanchez N, Rodriguez-Rodriguez A, Guerrero JM (2013). Melatonin: buffering the immune system. Int. J. Mol. Sci..

[40] Zwirner NW, Ziblat A (2017). Regulation of NK cell activation and effector functions by the IL-12 family of cytokines: the case of IL-27. Front. Immunol..

[41] Zhu W (2019). Integration of transcriptomics, proteomics and metabolomics data to reveal the biological mechanisms of abrin injury in human lung epithelial cells. Toxicol. Lett..

[42] Quiros PM (2017). Multi-omics analysis identifies ATF4 as a key regulator of the mitochondrial stress response in mammals. J. Cell. Biol..

[43] DeBerardinis RJ, Chandel NS (2016). Fundamentals of cancer metabolism. Sci. Adv..

[44] Zheng X (2019). Mitochondrial fragmentation limits NK cell-based tumor immunosurveillance. Nat. Immunol..

[45] Jadiya P, Tomar D (2020). Mitochondrial protein quality control mechanisms. Genes.

[46] Shi Y (2019). Gboxin is an oxidative phosphorylation inhibitor that targets glioblastoma. Nature.

[47] Liu X (2012). An artificial miRNA against HPSE suppresses melanoma invasion properties, correlating with a down-regulation of chemokines and MAPK phosphorylation. PLoS ONE.

