# Supplementary material for: Melatonin enhances radiofrequency-induced NK antitumor immunity, causing cancer metabolism reprogramming and inhibition of multiple pulmonary tumor development
Source: Signal Transduct Target Ther. 2021 Sep 1;6:330. doi: 10.1038/s41392-021-00745-7 (PMC8410827; doi:10.1038/s41392-021-00745-7)
Supplement: Supplementary file 1 — supplemental material [file 41392_2021_745_MOESM1_ESM.docx]

Supplementary Materials for

Melatonin enhances radiofrequency induced NK anti-tumor immunity, causing cancer metabolism reprogramming and inhibition of multiple pulmonary tumor development

Ming Li^1#^, Bingjie Hao^1#^, Menghuan Zhang^5#^, Russel J. Reiter^3^, Shumeng Lin^1^, Tiansheng Zheng^1^, Xiangyun Chen^1^, Yanbei Ren^1^, Liduo Yue^1^, Baigenzhin Abay^4^, Guojie Chen^1^, Xiao Xu^1^, Yufeng Shi^2^*, Lihong Fan^1^*

Correspondence to: Lihong Fan (fanlih@ aliyun.com), Yufeng Shi (yshi@tongji.edu.cn)

**This PDF file includes:**

Figures. S1 to S17

Tables S1 to S2

Figure. S1.


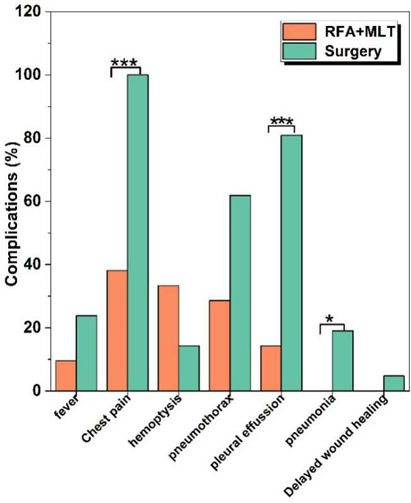


Complications of patients in RFA+MLT and surgery groups (*, *P* < 0.05; ***, *P* < 0.001).

Figure. S2.

**
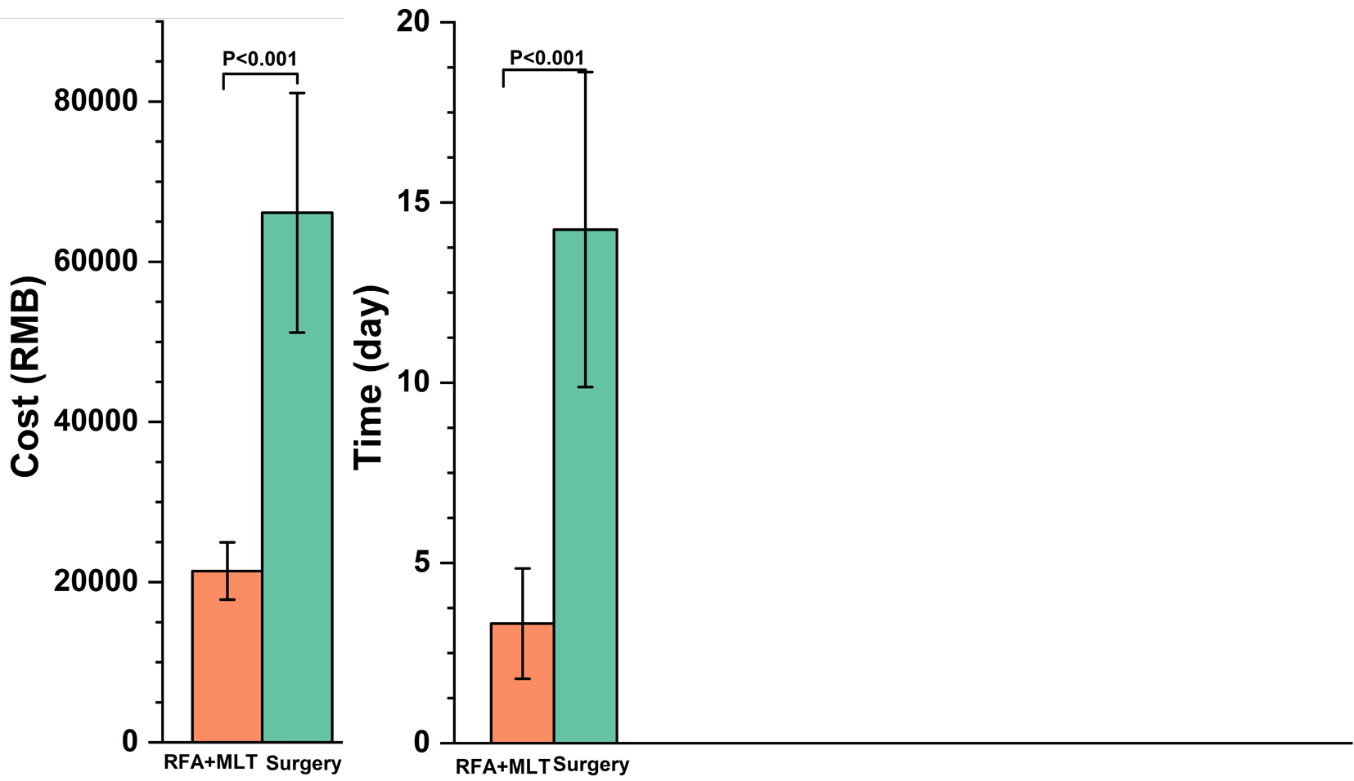
**

Cost and time spent in hospital for patients in RFA+MLT and surgery groups.

Figure. S3.


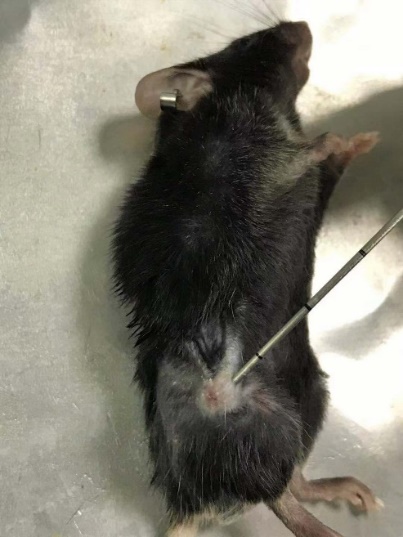


C57BL/6 mice with Lung cancer treated with RFA ablating on one side in RFA and RFA+MLT group.

Figure. S4.


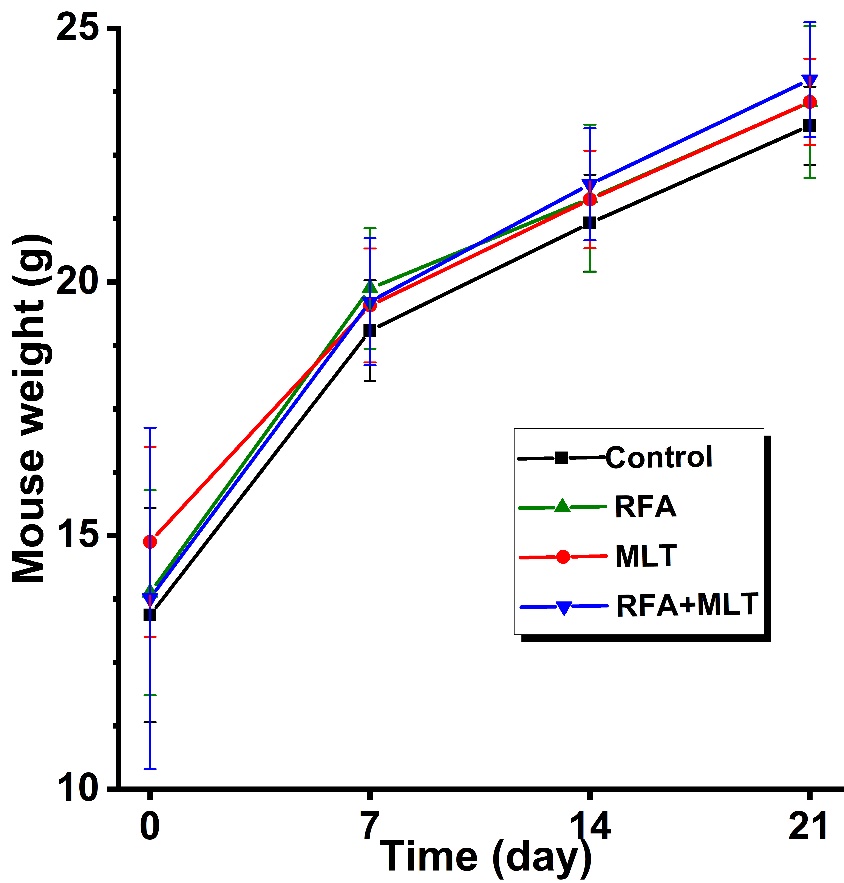


Mice weight after treated with RFA, MLT and RFA+MLT in different days, and the data showed no significance in statistical analysis.

Figure. S5.


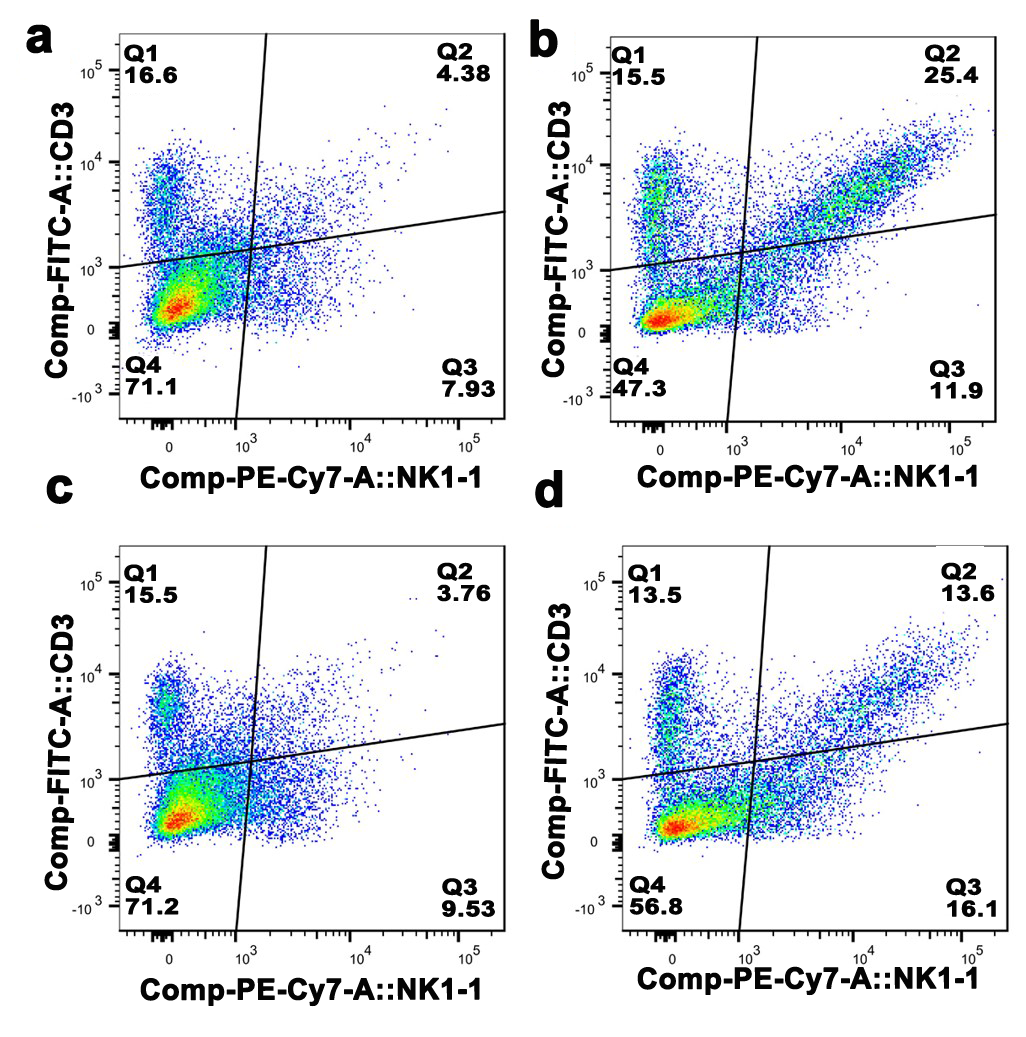


The flow cytometry of NK cells in C57BL/6 mice for (a). Control group, (b). RFA group, (c). MLT group and (d). RFA+MLT group.

Figure. S6.


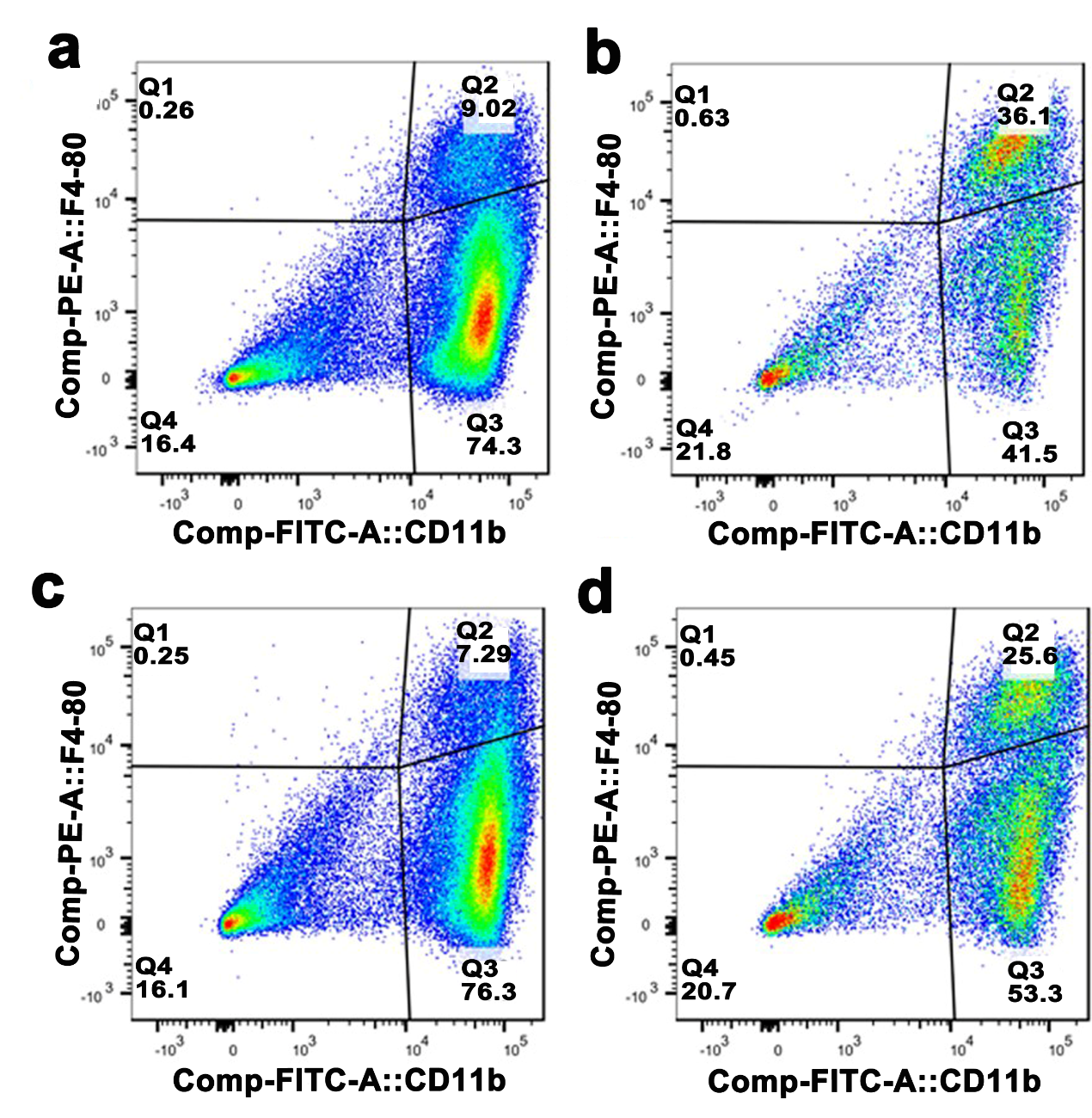


The flow cytometry of macrophage cells in C57BL/6 mice for (a). Control group, (b). RFA group, (c). MLT group and (d). RFA+MLT group.

Figure. S7.


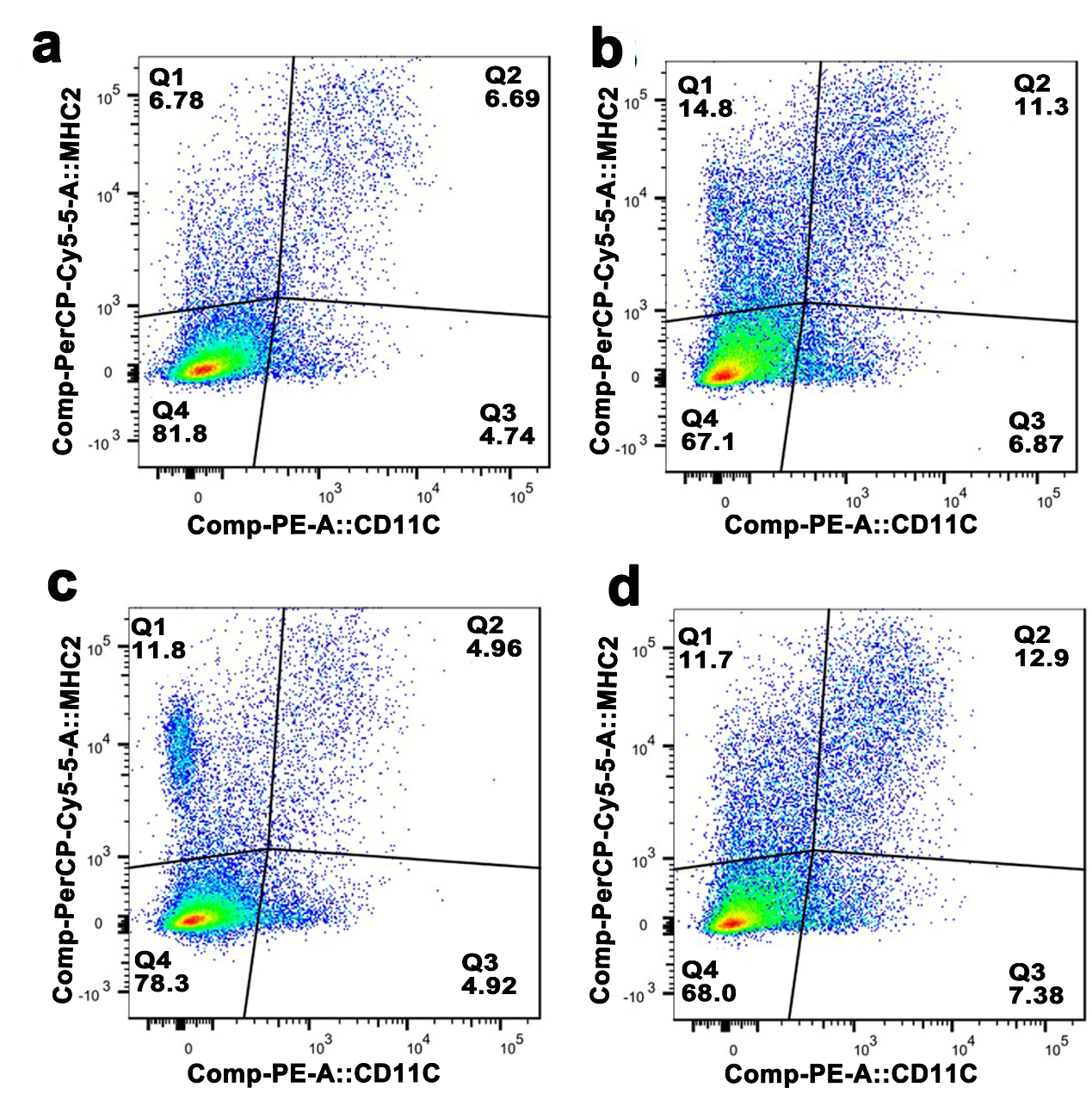


The flow cytometry of DC cells in C57BL/6 mice for (a). Control group, (b). RFA group, (c). MLT group and (d). RFA+MLT group.

Figure. S8.


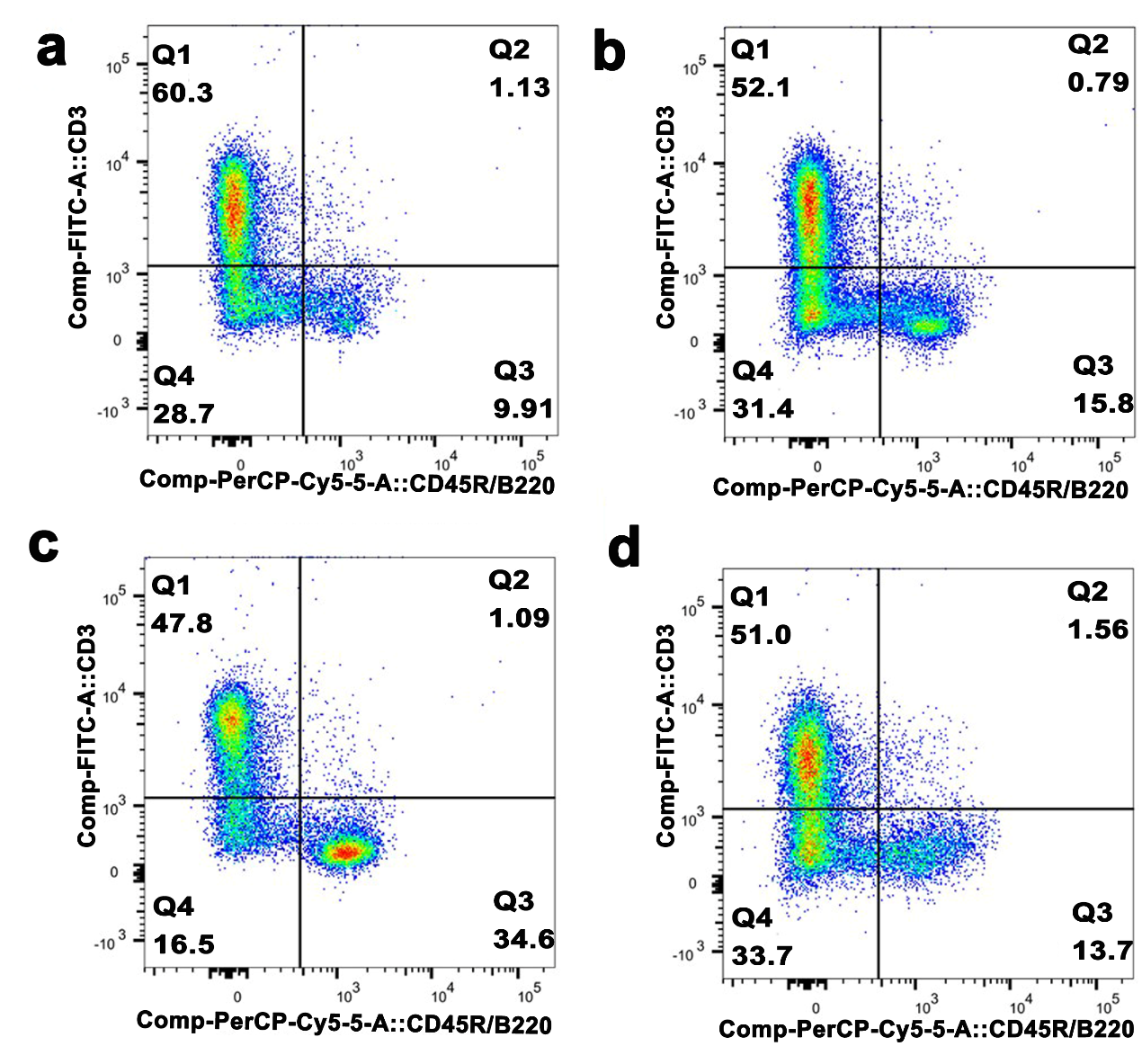


The flow cytometry of B cells in C57BL/6 mice for (a). Control group, (b). RFA group, (c). MLT group and (d). RFA+MLT group.

Figure. S9.


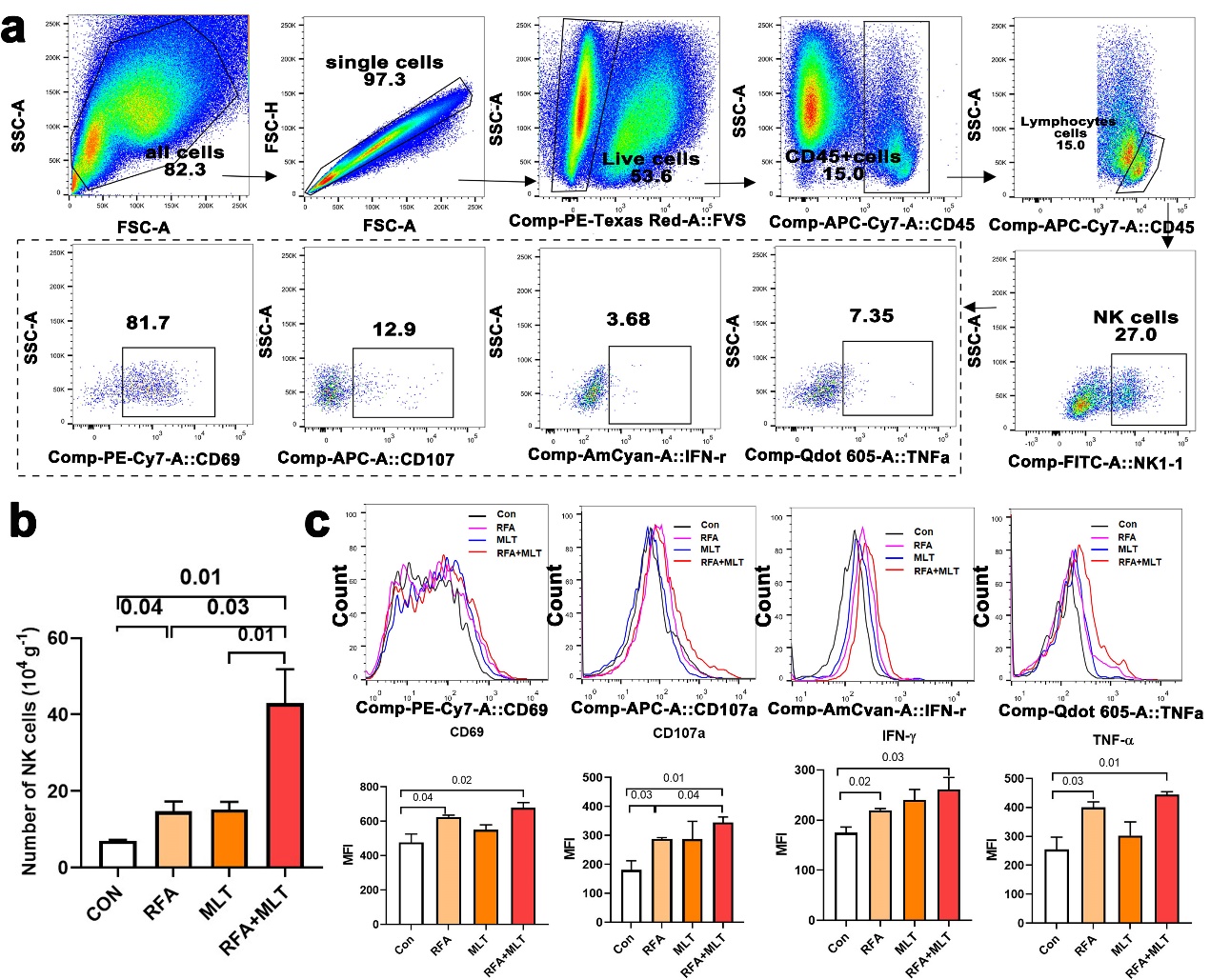


(a). The flow cytometry of CD45+/NK1.1+ cells and corresponding parameters (CD 69, CD 107a, IFN-γ and TNF-α) in C57BL/6 mice for Control group, RFA group, MLT group and RFA+MLT group. (b). Flow cytometry was used to analyze the numbers of CD45+/NK1.1+ cells in tumor tissue, for Control group, RFA group, MLT group and RFA+MLT group. (c). Flow cytometry was used to analyze the expression of CD 69, CD107 a, IFN-γ and TNF-α in CD45+/NK1.1+ cells in tumors, for Control group, RFA group, MLT group and RFA+MLT group.

Figure. S10.


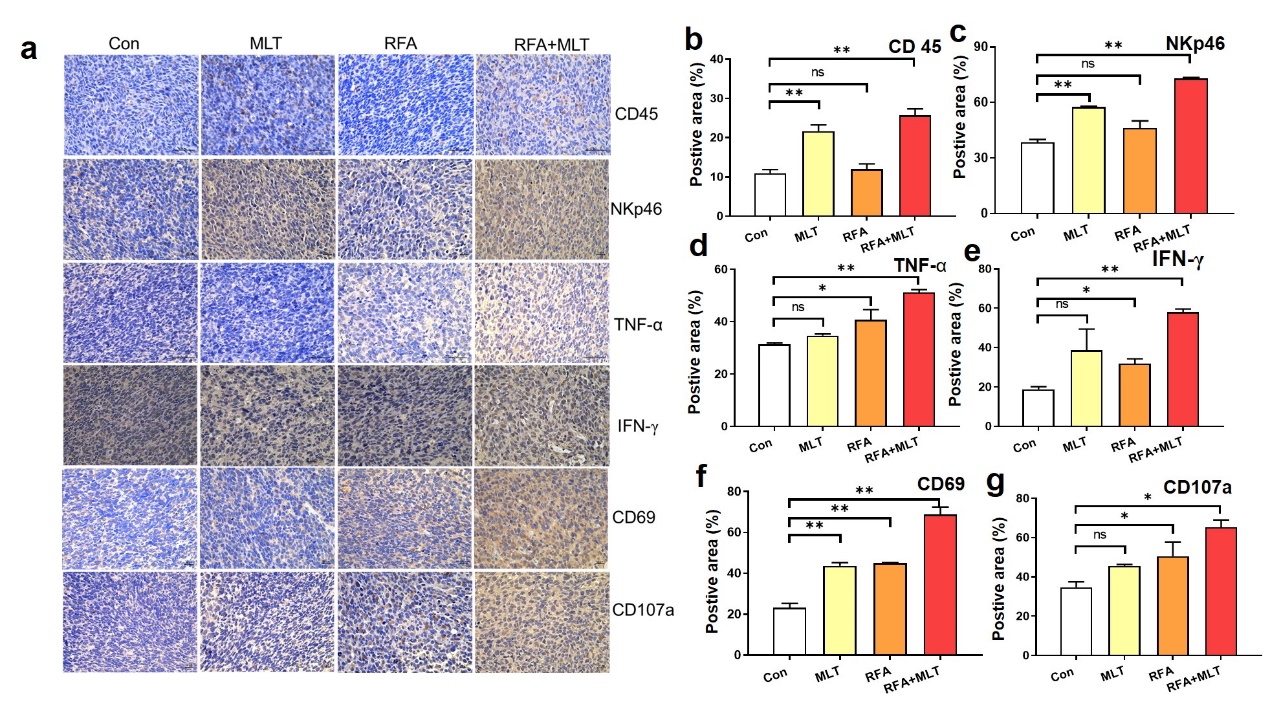


(a). Expression of CD 45, NKp46, TNF, IFN-γ, CD69 and CD107a in non-ablated tumor tissue samples via immunohistochemistry staining (IHC). The bar graph showed the level of (b). CD 45, (c). NKp46, (d). TNF-α, (e). IFN-γ, (f). CD69 and G. CD107a in non-ablated tumor tissue after treating with MLT, RFA and RFA+MLT, respectively (*, *P* < 0.05; **, *P* < 0 .01).

Figure. S11.


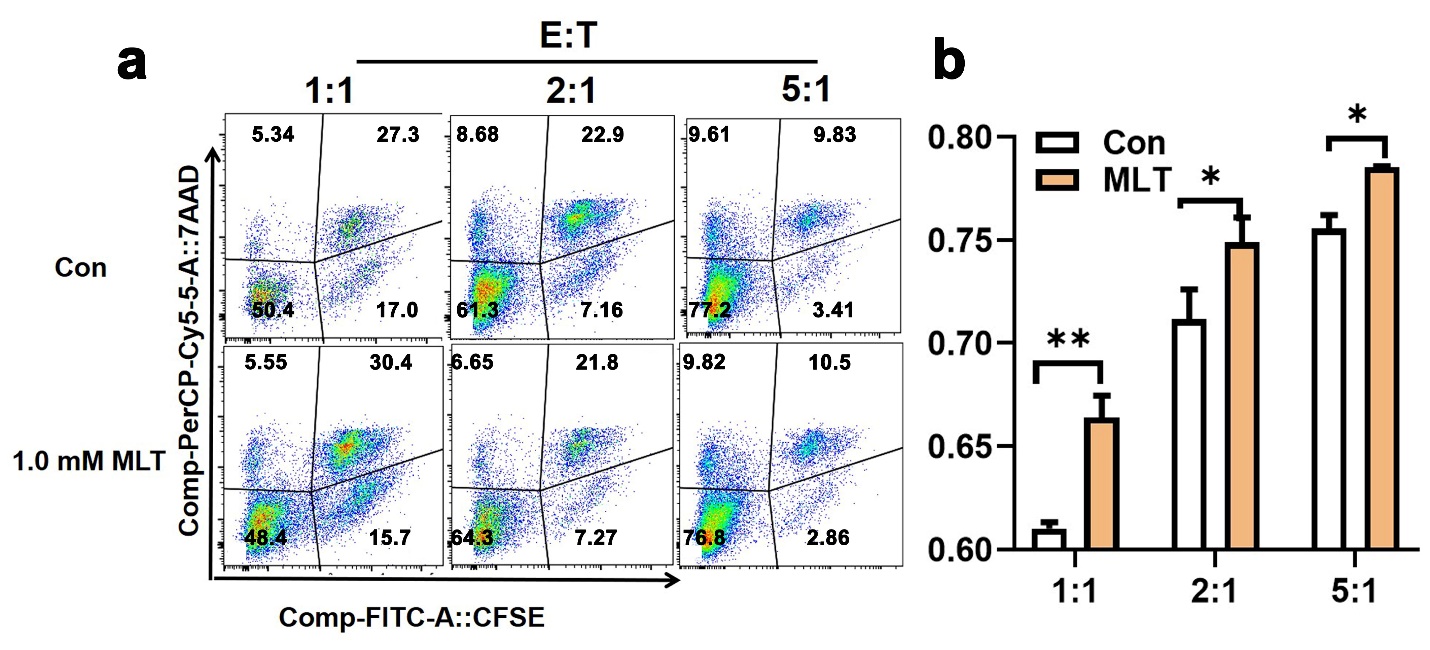


(a). *In vitro* cytotoxicity of NK92 cells in co-culture with K562 cells at the indicated E: T ratios (1:1, 2:1, 5:1), in which the NK92 cells were pre-treated with MLT (1.0 mM) for 48 h and the K562 cells were labeled with CFSE. (b). The graphs showed the apoptosis percentage of K562-CFSE via flow cytometry analysis (*, *P* < 0. 05; **, *P* < 0 .01).

Figure. S12.

The cell vitality of T cell after treated with different content MLT for 24 h (0 mM, 1 mM, 2 mM); the cell number were counted using Cellometer Auto T4 Cell Counter.

Figure. S13.


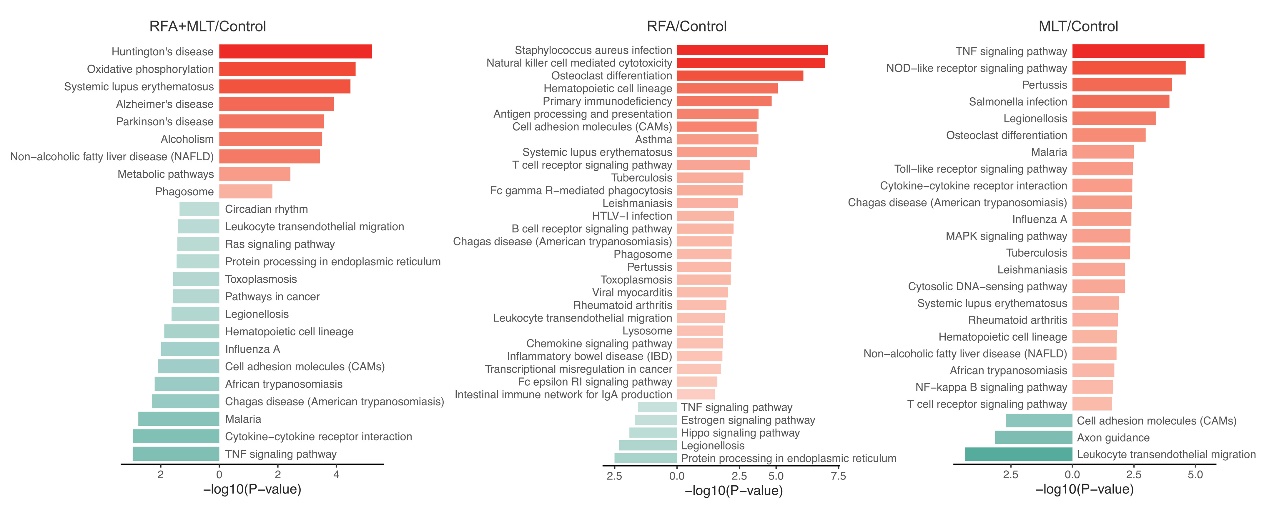


Pathway alterations in transcriptomic data. Alteration of pathways in non-ablated tumors after treated with MLT, RFA and RFA+MLT versus the Control group, respectively. Upregulated and downregulated pathways are indicated in red (right) and green (left) bars.

Figure. S14.


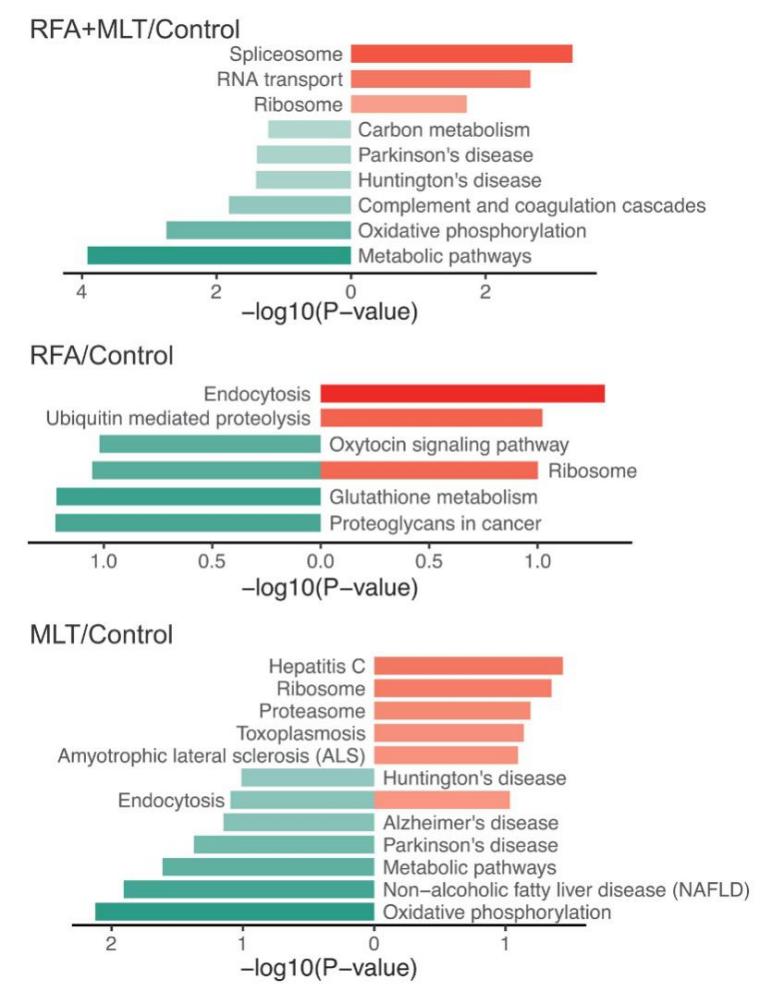


Pathway alterations in the proteomic data. Alteration of pathways in non-ablated tumors after treated with MLT, RFA and RFA+MLT versus the Control group, respectively. Upregulated and downregulated pathways are indicated in red (right) and green (left) bars.

Figure. S15.


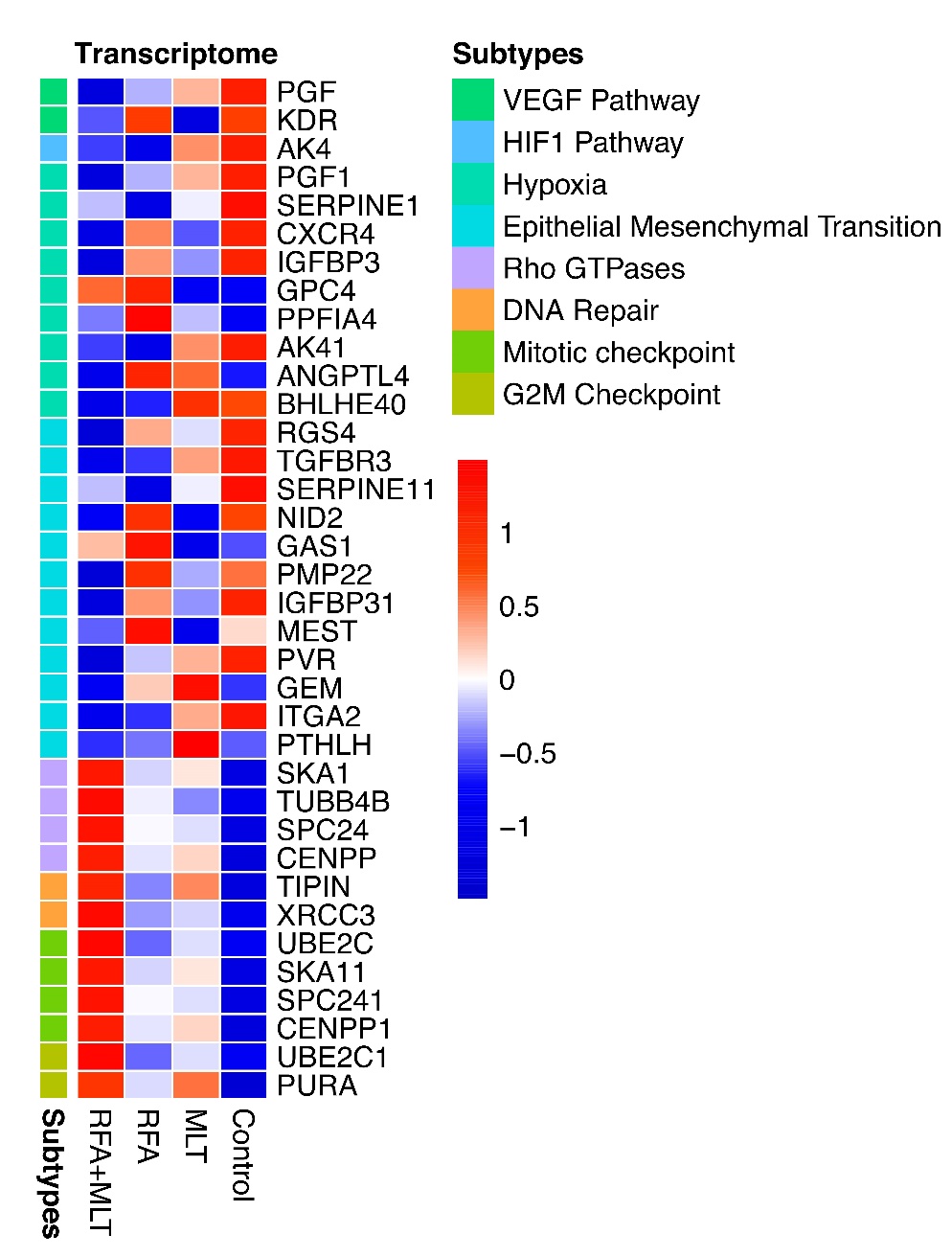


Heat map of alteration genes involved in the transcriptomic pathways shown in Figure 5a.

Figure. S16.


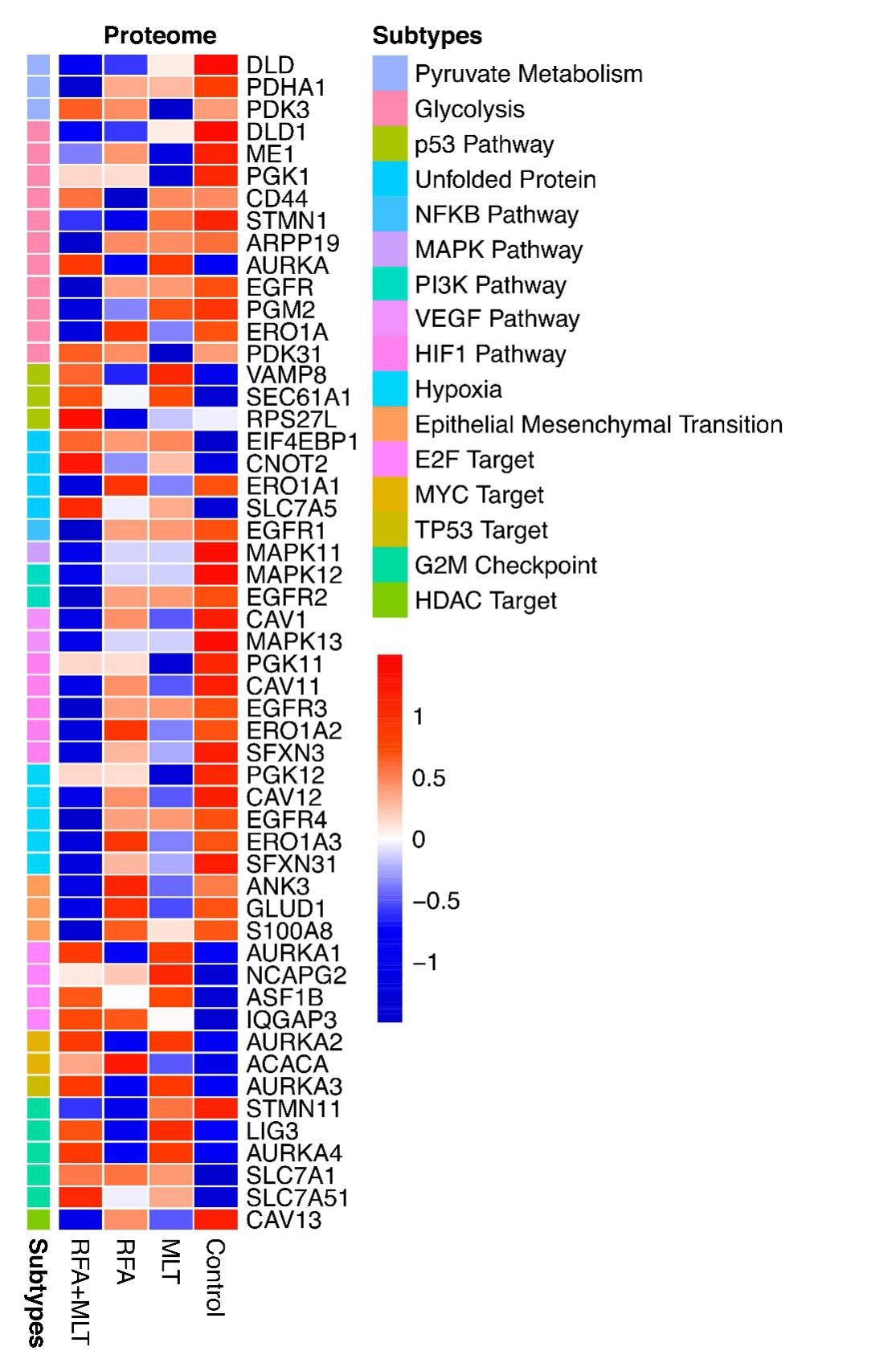


Heat map of alteration proteins involved in the pcroteomic pathways shown in Figure 5a.

Figure. S17.


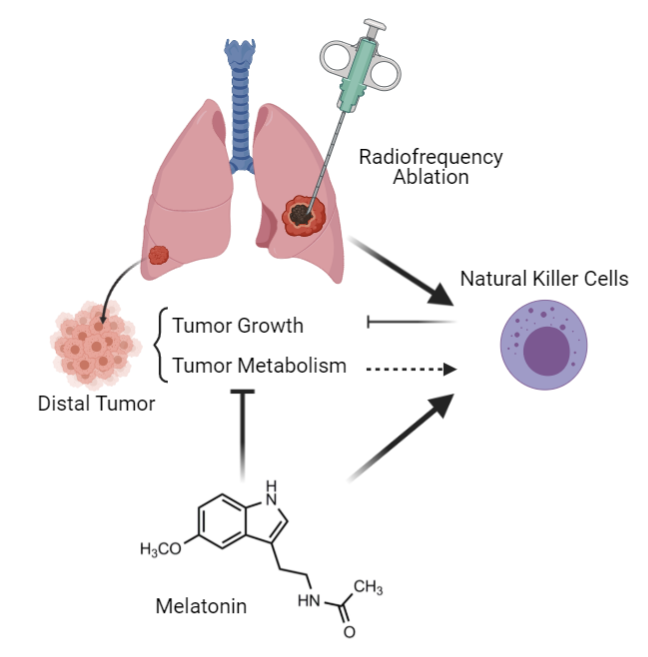


A schematic diagram illustrating the potential mechanism of the combination of RFA with MLT treatment.

Table S1.

Difference in lung function before and 12 months after treatment

in RFA+MLT and surgery groups

| **Index** | **RFA+MLT Group** | | |  | **Surgery Group** | | | ***P* value** |
| --- | --- | --- | --- | --- | --- | --- | --- | --- |
|  | **Before RFA**  **(n=21)** | **After RFA**  **(n=21)** | **Difference**  **(n=21)** |  | **Before Surgery**  **(n=21)** | **After Surgery (n=21)** | **Difference**  **(n=21)** |  |
| FEV_1_ (L) | 2.61(0.86) | 2.63(0.81) | 0.02(0.15) |  | 2.39(0.72) | 2.07(0.60) | -0.31(0.15) | <0.0001 |
| FEV_1_ (% of predicted value) | 95.72(15.99) | 96.95(14.79) | 1.22(5.06) |  | 88.13(7.25) | 76.77(5.14) | -11.36(4.70) | <0.0001 |
| VCMAX (L) | 3.16(0.88) | 3.14 (0.90) | -0.03(0.14) |  | 2.82(0.60) | 2.70(0.50) | -0.12(0.21) | 0.1101 |
| VCMAX (% of predicted value) | 93.95(11.51) | 93.10(13.67) | -0.83(4.16) |  | 88.19(8.57) | 84.62(7.59) | -3.56(6.01) | 0.0947 |
| FEV_1_/FVC% | 82.18(8.31) | 83.48(9.04) | 1.30(4.52) |  | 83.05(8.37) | 72.30(6.06) | -10.74(4.47) | <0.0001 |
| TLC (L) | 4.64(0.88) | 4.58(0.89) | -0.06(0.16) |  | 4.60(0.68) | 4.36(0.87) | -0.24(0.25) | 0.0083 |
| TLC (% of predicted value) | 86.92(7.83) | 85.75(8.76) | -1.17(2.99) |  | 88.28(9.57) | 83.45(13.39) | -4.82(4.94) | 0.0061 |
| MVV (L) | 96.62(22.34) | 96.40(20.78) | -0.22(4.87) |  | 90.84(21.83) | 83.83(18.83) | -7.01(5.75) | 0.0002 |
| MVV (% of predicted value) | 90.13(10.06) | 90.20(10.21) | 0.06(13.97) |  | 87.38(12.68) | 80.79(11.08) | -6.59(4.95) | 0.0463 |

Footnotes: 1. FEV_1_, forced expiratory volume in one second; 2. VCMAX, maximal vital capacity; 3. TLC, total lung capacity; 4. MVV, maximal vital volume.

Table S2.

Mouse-specific primer pairs used for quantitative reverse transcription PCR

| ID | Primer | RNA sequence | Base per |
| --- | --- | --- | --- |
| 1 | Hspa1b-F | GAGATCGACTCTCTGTTCGAGG | 22 |
| 2 | Hspa1b-R | GCCCGTTGAAGAAGTCCTG | 19 |
| 3 | Pgf-F | AGTGGAAGTGGTGCCTTTCAA | 21 |
| 4 | Pgf-R | GTGAGACACCTCATCAGGGTA | 21 |
| 5 | Fas-F | GCGGGTTCGTGAAACTGATAA | 21 |
| 6 | Fas-R | GCAAAATGGGCCTCCTTGATA | 21 |
| 7 | Angpt2-F | CAGCCACGGTCAACAACTC | 19 |
| 8 | Angpt2-R | CTTCTTTACGGATAGCAACCGAG | 23 |
| 9 | Cacna1g-F | TGTCTCCGCACGGTCTGTAA | 20 |
| 10 | Cacna1g-R | AAGCCGGTTCCAAGTGTCTC | 20 |
| 11 | Kit-F | GGCCTCACGAGTTCTATTTACG | 22 |
| 12 | Kit-R | GGGGAGAGATTTCCCATCACAC | 22 |
| 13 | Hspa1a-F | TGGTGCAGTCCGACATGAAG | 20 |
| 14 | Hspa1a-R | GCTGAGAGTCGTTGAAGTAGGC | 22 |
| 15 | Map2k6-F | GATGACCTGGAGCCGATAGTG | 21 |
| 16 | Map2k6-R | ATCAGCAGCCGTTTCTGTTCC | 21 |
| 17 | Dusp4-F | TCCCCGTCGAAGACAACCA | 19 |
| 18 | Dusp4-R | CTTTACTGCGTCGATGTACTCG | 22 |
| 19 | Sox17-F | GATGCGGGATACGCCAGTG | 19 |
| 20 | Sox17-R | CCACCTCGCCTTTCACCTTTA | 21 |
| 21 | Mdm4-F | TTCGGAACAAATTAGTCAGGTGC | 23 |
| 22 | Mdm4-R | GTGCATTACCTCTTTCATGGTGA | 23 |
| 23 | Siva1-F | CGCCCATCGCTTGTTCATC | 19 |
| 24 | Siva1-R | CTCACCATCGTCGGCATAGTC | 21 |
| 25 | Igfbp3-F | CACACCGAGTGACCGATTCC | 20 |
| 26 | M-IL-1b-F | GAACATGCCACCTTTTGACAGTG | 22 |
| 27  28  29  30 | M-IL-1b-R  M-Ptgs2-F  M-Ptgs2-R  M-Cxcl1-F | TGGATGCTCTCATCAGGACAG  TTCCAATCCATGTCAAAACCGT  AGTCCGGGTACAGTCACACTT  ACTGCACCCAAACCGAAGTC | 21  22  21  20 |
| 31  32  33 | M-Cxcl1-R  GAPDH-F  GAPDH-R | TGGGGACACCTTTTAGCATCTT  AGGTCGGTGTGAACGGATTTG  CTTCCCATTCTCGGCCTTG | 22  21  19 |
